# Protein disorder-to-order transition enhances the nucleosome-binding affinity of H1

**DOI:** 10.1093/nar/gkaa285

**Published:** 2020-05-01

**Authors:** Akshay Sridhar, Modesto Orozco, Rosana Collepardo-Guevara

**Affiliations:** 1 Maxwell Centre, Cavendish Laboratory, University of Cambridge, JJ Thomson Avenue, Cambridge CB3 0HE, UK; 2 Institute for Research in Biomedicine, The Barcelona Institute of Science and Technology, Baldiri i Reixac, 19, 08028 Barcelona, Spain; 3 Department of Biochemistry and Biomedicine, University of Barcelona, Av. Diagonal 647. 08028 Barcelona, Spain; 4 Department of Chemistry, University of Cambridge, Lensfield Road, Cambridge CB2 1EW, UK; 5 Department of Genetics, University of Cambridge, Cambridge CB2 3EH, UK

## Abstract

Intrinsically disordered proteins are crucial elements of chromatin heterogenous organization. While disorder in the histone tails enables a large variation of inter-nucleosome arrangements, disorder within the chromatin-binding proteins facilitates promiscuous binding to a wide range of different molecular targets, consistent with structural heterogeneity. Among the partially disordered chromatin-binding proteins, the H1 linker histone influences a myriad of chromatin characteristics including compaction, nucleosome spacing, transcription regulation, and the recruitment of other chromatin regulating proteins. Although it is now established that the long C-terminal domain (CTD) of H1 remains disordered upon nucleosome binding and that such disorder favours chromatin fluidity, the structural behaviour and thereby the role/function of the N-terminal domain (NTD) within chromatin is yet unresolved. On the basis of microsecond-long parallel-tempering metadynamics and temperature-replica exchange atomistic molecular dynamics simulations of different H1 NTD subtypes, we demonstrate that the NTD is completely unstructured in solution but undergoes an important disorder-to-order transition upon nucleosome binding: it forms a helix that enhances its DNA binding ability. Further, we show that the helical propensity of the H1 NTD is subtype-dependent and correlates with the experimentally observed binding affinity of H1 subtypes, suggesting an important functional implication of this disorder-to-order transition.

## INTRODUCTION

In Eukaryotic cells, genomic DNA is packaged into chromatin, a nucleoprotein complex whose fundamental repeating unit is the nucleosome (1,2). Nucleosomes are formed by ∼147 bp of DNA wrapped around a histone protein octamer core (two copies each of H3, H4, H2A and H2B) and are joined together by free DNA linker segments. Most organisms contain an additional type of histone, the H1 linker histone protein. H1 is a chromatin architectural protein that binds to nucleosomes near the entry/exit site of the linker DNA (3,4), interacts with ∼20 bp of the DNA linkers (5), and critically influences chromatin organization (6–8).

The family of H1 proteins is highly divergent (9). In mammals, up to 11 different H1 subtypes (H1.1–H1.10 and H1.0) have been identified (10–12) and characterized as having differing expression timings (13), extent of post-translational modifications (11,14), chromatin compacting capabilities (15), preference for eu-/heterochromatin regions (16), and interactions with other chromatin architectural proteins (17,18).

**Figure 1.**
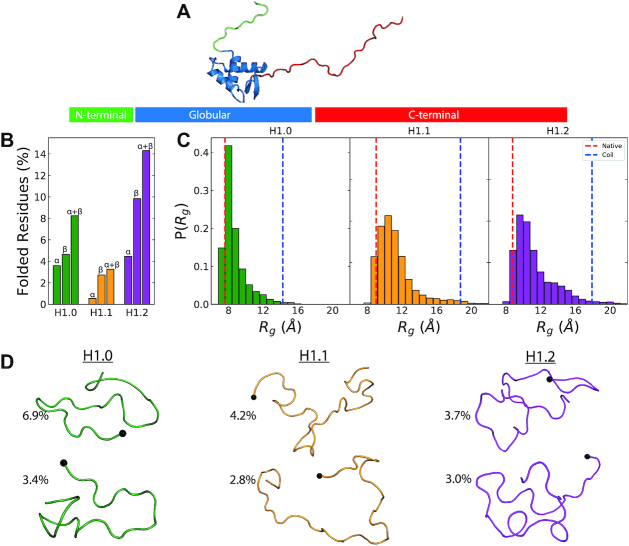
Structure of H1 (**A**) The constituent domains of H1 – winged-helix globular domain (blue), unstructured NTD (green) and unstructured CTD (red). (**B**) Ensemble averages of the percentage of secondary structures in each of the three NTDs from the final 200 ns of the 300 K T-REMD trajectory. (**C**) Distributions of the radius of gyration (*R*_g_) of the NTD Cα atoms. The experimentally predicted regressions for random-coil and globular proteins are shown in blue and red respectively. (**D**) Illustrations of the highest populated clusters of the three NTDs. The final residue of the NTDs (point of attachment to the winged-helix globular domain) is shown as a black sphere.

From the protein structure point of view, the H1 proteins contain a ∼75 residue-long winged-helix globular domain, an unstructured N-terminal domain (NTD) of up to 45 residues, and a long mostly unstructured C-terminal domain (CTD) of ∼100 residues (19,20) (Figure 1A). The globular domain is highly conserved within the H1 family (21,22) but the NTD and CTD vary significantly in sequence and length. This has led to the hypothesis that the differential behaviour among H1 subtypes stems from the heterogeneity in the diverging unstructured terminal domains (14). Because the CTD is believed to account for most of the subtype heterogeneity (23–25), significant efforts have been made to understand the conformational landscape of the CTD in isolation (26), when bound to DNA (27) or to a negatively charged IDP with high affinity (28), and when bound to a full chromatosome (4,29).

H1's shortest domain, the NTD, plays a negligible role in H1's chromatin compaction capability (30). However, its deletion reduces the binding affinity of H1 to the nucleosome significantly (30,31). Across H1 subtypes, an analysis of the NTD role has shown contrasting results with swaps of the H1.0/H1.2 and H1.0/H1x NTDs resulting in an exchange of their binding characteristics (32,33), but swaps of H1.1/H1.5 NTDs displaying no effects (23). The NTDs are rich in basic residues crucial for DNA interactions and otherwise predominantly composed of G/E/P/S/T/A amino acids – an archetypical composition of IDPs (34). Indeed, the NTD has been shown to be unstructured in aqueous solution and to have a propensity to fold in the presence of short DNA segments (35). However, whether the NTD remains disordered or undergoes a disorder-to-order transition when H1 binds to the nucleosome is not known, which limits our molecular mechanistic understanding of its functional role (36,37).

Given the unstructured and flexible nature of the NTD, characterizing its conformational landscape experimentally at atomistic resolution is challenging. Complementary to the experimental efforts, advanced simulation methods, such as enhanced sampling molecular dynamics (MD) simulations, have been successfully used in recent years to investigate the configurational ensembles of core histone tails (38,39) and the CTD of H1 (29), and to understand the role of post-translational modifications within them (40,41). Here, we take advantage of recent advances in enhanced-sampling methods and the development of state-of-the-art biomolecular force fields (42,43) for the description of IDPs. In particular, we use the Parallel-Tempered Metadynamics in the Well-Tempered Ensemble (PTMetaD-WTE) method (44)—a combination of the Temperature Replica-Exchange (T-REMD) and metadynamics techniques developed to facilitate sampling of phase space. While T-REMD assists in the crossing of high energy barriers via high temperature replicas, MetaD-WTE accelerates sampling through the use of a history-dependent biasing potential acting along specific collective variables (CV) (see Materials and Methods). The combination of both helps overcome two well-known limitations of using T-REMD or metadynamics independently: (1) thorough sampling along slow diffusing CVs not considered explicitly (44), and (2) increase of the energetic overlap among temperature replicas and, hence, significant reduction of the total number of replicas needed and the associated computational costs (45) (see Materials and Methods).

By means of a large set of microsecond-long PTMetaD-WTE, T-REMD, and Biased-Exchange Metadynamics (BEMD) simulations (Supplementary Table S1), we fully characterize the difference in structure across various NTD subtypes. We compare how the structural behaviour of the NTD changes when it is free in solution, versus when it is bound to DNA or to a full 211-bp nucleosome (a 147-bp nucleosome with two symmetric 32-bp linker DNA arms). Our results show that the NTD of H1 is completely unstructured in solution, but upon DNA or nucleosome binding transitions into an amphiphilic helix with charged and uncharged residues on opposing faces. Further, we demonstrate that formation of this amphiphilic helix depends on the charge neutralization of the Lys sidechains by DNA. Crucially, our work demonstrates that the amphiphilic helical propensity of the NTD of H1 varies proportionally to the experimentally observed binding affinity of H1 subtypes, suggesting a potential implication of the NTD disorder-to-order transition in the function of linker histones.

## MATERIALS AND METHODS

### System and simulation setup

The sequences of the human H1 subtypes were obtained from the Uniprot Database (46) and their N-terminal domains were built in an extended conformation using Avogadro (47) without capping of the N- or C-terminus. For computational expedience associated with reducing the solvent molecules required, we bent the initial linear configurations through a short 5 ns simulation in GBSA implicit solvent (39,48). The resulting structures were then used as initial configurations for all further simulations. The basic sub-region of each NTD was defined as commencing three residues (one-turn) before the first Lys of the NTD.

The simulations were performed using Gromacs 2016 (49) with Plumed 2.3.0 (50) in explicit solvent and 0.15 M Na^+^/Cl^−^ ions. Two state-of-the-art force fields were used for the T-REMD simulations: Charmm36M (42), and Amber99SB-ILDN (51) with Helix-Coil transition specific backbone (52) and charge (53) corrections (Amberff99sb*-ildn-q). With the Amber force field, water was modelled using the TIP3P parameters (54), and ions with the parameters of Aqvist (55) and Dang (56). With Charmm36M, water was modelled using the modified TIP3P parameters with increased Hydrogen atom Lennard-Jones well-depth (42), and ions with the parameters of Beglov and Roux (57), including the nbfix changes of Luo and Roux (58) and Venable *et al.* (59).

For the 20-bp B-DNA strands, we used two different force fields: Charmm DNA and parmbsc0 (60,61). The Settle algorithm (62) was used to constrain bond lengths and angles of water molecules and P-LINCS was used for all other bond lengths. Temperatures were maintained using the v-rescale thermostat (63) and the pressure at 1 bar using the Parrinello-Rahman barostat (64). Long range electrostatic interactions were calculated using the Particle Mesh Ewald (PME) (65) algorithm with a cut-off of 1.0 nm.

### Temperature replica-exchange simulations

Temperature Replica-Exchange (T-REMD) simulations were performed starting from representative NTD configurations obtained through implicit-solvent equilibration. A temperature range of 300–450 K was used and the number/temperature of replicas were calculated using the predictor of Patriksson and van der Spoel (66) and an acceptance probability of 20%. This resulted in 56, 96 and 72 replicas for the H1.0, H1.1 and H1.2 subtypes respectively. The replicas were simulated for 250 ns each and exchanges between adjacent temperatures were attempted every 10 ps. The trajectory frames from the lowest temperature (300 K) were then used for analysis. Convergence of the T-REMD simulations were assessed by monitoring changes in the helical and beta content of the residues in 40 ns intervals after discarding the initial 50 ns for equilibration.

### Parallel-tempered metadynamics in the well-tempered ensemble

We begin by applying a time-dependent biasing potential, V(S,t), defined as a sum of Gaussians deposited along the collective variables (CV) space (*S*)

$$\begin{equation*}V\left( {S,t} \right)\ = \ W\mathop \sum \limits_{t^{\prime}\ = {\tau _{G,}}\ 2{\tau _G} \ldots } \exp\left( { - \frac{{{{\left( {S - {S_{t^{\prime}}}} \right)}^2}}}{{2{\sigma ^2}}}} \right)\end{equation*}$$

where τ_G_ is the time interval at which the Gaussians are added with height *W*, width σ and mean *S*_*t*’_. The biasing potential discourages the trajectory from visiting states already sampled. Choosing an accurate set of CVs to adequately promote sampling is often non-trivial (45) with energy barriers along unaccounted CVs affecting sampling efficiency (67). The Parallel-Tempered Metadynamics (PTMetaD) (68) method combines the above described T-REMD with metadynamics to deal with unaccounted slow degrees of freedom. The metadynamics biases of individual replicas at different temperatures are evolved independently and the adjacent replicas can exchange with a probability of

$$\begin{eqnarray*} P &=& {\rm min} \left\{1, \exp \left[({\rm \beta }_i - {\rm \beta}_j)(U_i - U_j) \right. \right.\\ &&+ \left. \left. {\rm \beta }_i\left(V_i(S_i) - V_i(S_j) \right) + {\rm \beta }_j\left(V_j(S_j) - V_j(S_i) \right) \right] \right\} \end{eqnarray*}$$

where *U* is the potential energy, *V* is the bias potential and β is the inverse of the thermodynamic temperature (1/*k*_B_*T*).

However, energy distributions of the replicas *‘i’* and *‘j’* need to sufficiently overlap for exchanges to occur (Supplementary Figure S1). This in-turn requires the use of a large number of replicas as predicted for the T-REMD simulations. Here, we use the Well-Tempered Ensemble (WTE) (44) to improve efficiency. The method enhances energetic fluctuations of each replica and thereby its overlap with adjacent replicas through the use of the potential energy as an independent CV. For the current work, we used the procedure of Bernetti *et al.* (69) with eight temperatures geometrically distributed between 298 and 400 K as

$$\begin{equation*}\ {T_i} = {T_{{\rm min}}}\ {\left( {\frac{{{T_{{\rm max}}}}}{{{T_{{\rm min}}}}}} \right)^{\left( {i - 1} \right)/\left( {N - 1} \right)}}\end{equation*}$$

where *N* is the number of replicas. The configurations obtained from implicit solvent equilibration were equilibrated in the eight temperatures before a preliminary 10 ns PTMetaD-WTE run was carried out. This initial run used only the potential energy (U) as the CV and deposited Gaussians of height 2.5 kJ/mol, width 500 kJ/mol and bias factor of 50. The Gaussians were deposited every 250 steps and sufficient overlap of the replica potential energies was ensured through a histogram analysis at the end of this run (see Supplementary Figure S1).

The bias from this preliminary run was then kept fixed in the production PTMetaD-WTE simulation and ensured exchanges between adjacent replicas. This production run used two CVs: (1) the alpha helical content (*S*_α_) and (2) the radius of gyration (*S*_rg_) of Cα atoms. The alpha helical content CV (*S*_α_) has been used extensively as a CV within metadynamics simulations to sample the random coil-helix transformation of proteins, with back-calculated NMR shifts of the resulting conformational ensembles being in good agreement with experimental values (69–71). It is defined following the work of Pietrucci and Laio (72) as

$$\begin{equation*}S{\ _{\rm{\alpha }}} = \sum \frac{{1 - {{\left( {\frac{{{\rm{\Delta }}{\rm RMSD}}}{{{R_0}}}} \right)}^n}}}{{1 - {{\left( {\frac{{{\rm{\Delta }}{\rm RMSD}}}{{{R_0}}}} \right)}^m}}}\ \end{equation*}$$

where *R*_0_, *n* and *m* are 0.08 nm, 8 and 12 respectively. ΔRMSD is the root mean square difference of six residue segments between the configuration and ideal alpha conformations. The Backbone (Cα, C, N, O) and Cβ atoms were included in the RMSD calculation.

The radius of gyration CV (*S*_rg_) of the Cα atoms is defined as

$$\begin{equation*}{\rm{\ }}{S_{rg}} = {\left( {\frac{{\mathop \sum \nolimits_i^n {m_i}{{\left| {{r_i} - {r_{com}}} \right|}^2}}}{{\mathop \sum \nolimits_i^n {m_i}}}} \right)^{1/2}}{\rm{\ }}\end{equation*}$$

where *r_i_* and *m_i_* are the positions of and mass of Cα atom *i. r_com_* is the centre of mass of the NTD Cα atoms.

### DNA–protein metadynamics simulations

The H1.0 subtype was used to examine the effects of DNA binding on the conformational preferences of the NTD. The nucleosomal on-dyad structure of the H1.0 globular domain (PDB ID: 5NL0 (4)) was obtained and Modeller (73) was used to add the NTD basic subregion in an extended random coil configuration. The relative orientations of this built basic subregion and one of the nucleosomal linker arms (20 bp) was used as the initial configuration (Figure 3A) for metadynamics simulation.

The metadynamics simulations utilized two CVs: (1) the alpha helical content (*S*_α_) and (2) the number of NTD-DNA electrostatic contacts (*S*_cont_). The CVs thus simultaneously allow exploring NTD–DNA binding/unbinding and changes in helical propensity. The *S*_cont_ CV was defined as

$$\begin{equation*}\ {S_{{\rm cont}}} = \ \mathop \sum \limits_{i \in {H^ + }} \mathop \sum \limits_{j \in {O^ - }} \frac{{1\ - \ {{\left( {\frac{{\overrightarrow {{r_i}} - \overrightarrow {{r_j}} }}{{{R_0}}}} \right)}^n}}}{{1\ - \ {{\left( {\frac{{\overrightarrow {{r_i}} - \overrightarrow {{r_j}} }}{{{R_0}}}} \right)}^m}}}\end{equation*}$$

where *R*_0_, *n* and *m* are 0.2 nm, 8 and 10 respectively. H^+^ is the set of all positively charged sidechain hydrogen atoms within Lys/Arg residues and O^−^ is the set of backbone oxygen atoms within the central 11-bp of DNA. Metadynamics gaussians were deposited every 10 ps and the simulation was run for 600 ns and the initial 50 ns was disregarded for analysis. To assist convergence, the lowest terminal base-pair (BP 20) and the backbone of the NTD’s carboxy-terminal residue were both restrained with a force constant of 750 kJ/mol/nm^2^.

### Trajectory analysis

The simulation trajectories were analysed using a combination of Plumed/Gromacs tools together with Python scripts using the MDAnalysis and MDTraj libraries (74,75). The secondary structures were assigned using DSSP (76) with residues being part of either alpha- or 3_10_-helix motifs counted towards the total helical propensity. The theoretical compaction (radius-of-gyration) of proteins with globular (77) and random-coil (78) structures were calculated as

$$\begin{equation*}R_g^{glob}\left( N \right)\ = \ 2.2{N^{0.38}}\end{equation*}$$

$$\begin{equation*}R_g^{coil}\ (N) = \ 2.02{N^{0.60}}\end{equation*}$$

Clustering was performed using the single-linkage clustering method where the structure ‘*j’* is assigned to cluster ‘*i*’ if the RMSD of Cα atoms between them is < 2.5 Å.

In the metadynamics simulations, the addition of history dependent bias potentials precludes a direct ensemble averaging of the system's characteristics as simulation time is without physical meaning. The methodology of Tiwary and Parrinello (79) was thus used to reweight trajectory frames and subsequently calculate the unbiased equilibrium averages. Briefly, the Probability Distribution of the biased system *P*(*R,t*) can be expressed as

$$\begin{equation*}P\ \left( {R,t} \right) = \frac{{\exp \left( { - {\rm{\beta }}\left[ {U\left( R \right) + V\left( {s\left( R \right),t} \right)} \right]} \right)}}{{\smallint \exp \left( { - {\rm{\beta }}\left[ {U\left( R \right) + V\left( {s\left( R \right),t} \right)} \right]} \right)dR}}\ \end{equation*}$$

where *U*(*R*) is the internal potential and *V*(*s*(*R*)*,t*) is the bias potential as given by Equation (1). The introduction of the delta function *δ*(*s – S*(*R*)) and the unbiased probability density function *P*_0_(*R,t*) allows the expression of equation (7) as

$$\begin{equation*}P\ \left( {R,t} \right) = {P_0}\ \left( {R,t} \right) \cdot \exp \left( { - {\rm{\beta }}\left[ {V\left( {s\left( R \right),t} \right) - c\left( t \right)} \right]} \right)\end{equation*}$$

where *c*(*t*) is the time-dependent bias offset (80) defined as

$$\begin{equation*}c\ \left( t \right) = \frac{1}{{\rm{\beta }}}\ \ln \left[ {\frac{{\smallint \exp \left( { - {\rm{\beta }}F\left( s \right)} \right)ds}}{{\smallint \exp \left( { - {\rm{\beta }}\left[ {F\left( s \right) + V\left( {s,t} \right)} \right]} \right)ds}}} \right]\end{equation*}$$

The *c*(*t*) offset is calculated using Plumed following the protocol of Tiwary and Parrinello (79) and used to assign weights $w( t ) \propto \exp ( {{\rm{\beta }}[ {V( {s( R ),t} ) - c( t )} ]} )$ from the biased probability density. See Bonomi *et al.* (80) for a complete derivation. The equilibrium average of any system characteristic *O* is then calculated as

$$\begin{equation*} {\langle O \rangle} = \frac{{\sum {w_i}\left( t \right){O_i}}}{{\sum {w_i}}}\ \end{equation*}$$

For the calculation of per-residue helical content, per-frame *O_i_* of the residue was set as 1 if DSSP (76) predicted an alpha/3_10_ motif and 0 otherwise. Similarly, the clusters were reweighted using *w*(*t*) of the trajectory frames assigned to them.

### Docking

Docking of the clustered conformations from the PTMetaD-WTE simulations were performed using the HADDOCK webserver (81). The B-DNA for docking was built as a 20-bp long strand using the Nucleic Acids Builder within Amber16 (82). To allow for all possible orientations of the NTD basic sub-regions when interacting with this strand, the central 12-bp (one-turn) were considered the ‘active’ residues for docking. For the NTD basic subregions, the Lys residues within the basic ‘face’ were considered the ‘active’ residues. The His residues were considered neutrally charged and no segment within the DNA or NTD was considered flexible.

### Potential of mean force calculations

The Potential of Mean Force (PMF) calculations were performed using a protocol similar to that of Wieczor and Czub (83). Firstly, the helical axis of the 20-bp strand was aligned along the z-axis and the radial-distance between the Central BP and the Helix Cα atoms along the XY-plane was used as the CV. Initial frames for the umbrella windows were generated from the docked configuration by increasing this radial distance CV using 20 windows at intervals of 0.1 nm. Each window was then simulated for 100 ns using an umbrella biasing potential of 750 kJ/mol/nm^2^ along the CV. To reduce diffusion of the DNA strand and thereby assist convergence (84), the two terminal base-pairs at each end (BP 1,2,19,20) were restrained with a force constant of 500 kJ/mol/nm^2^. The initial 25 ns were discarded, and the rest of the trajectory was used for analysis. PMF profiles were generated using the GROMACS implementation (g_wham) (85) of the weighted histogram analysis method (WHAM). Bayesian bootstrapping with 100 bootstraps were used to estimate the errors for each profile.

## RESULTS AND DISCUSSION

### The isolated H1 NTD peptide is disordered regardless of subtype

The amino acid sequences of the various H1 NTD subtypes display several distinguishable features of IDPs: low hydrophobicity, high net charge, and low sequence complexity (86). Thus, we first determine if the varying amino acid sequences of the different subtypes of the H1 NTD are sufficient to induce different structural behaviours, such as, random coil conformations, disordered structures with flickering secondary structural elements, or stable secondary structural folds. To answer this, we assess the conformational landscape of the isolated NTD peptides of three different H1 human subtypes—H1.0, H1.1 and H1.2—in solution through T-REMD simulations. We consider temperature replicas ranging from 300 to 450 K and run each replica for 250 ns for an accumulated sampling of up to 24 μs per system (see Materials and Methods).

We find that for all subtypes, the H1 NTD emerges as a highly unstructured domain displaying only transient secondary structural elements (Figure 1), irrespective of the force field used (Amberff99sb*-ildn-q (51–53) and Charmm36M (42); Supplementary Figure S2). Indeed, for both force fields, the conformational ensembles include only a small fraction (<15%) of residues with α-helical or β-strand structural elements (Figure 1B). These observations are consistent with circular dichroism experiments (35,87) showing that the spectra for the NTDs of H1.0 and mH1.4 (homologous to hH1.2, Supplementary Figure S3) in aqueous solution are dominated by contributions of the random coil.

**Figure 2.**
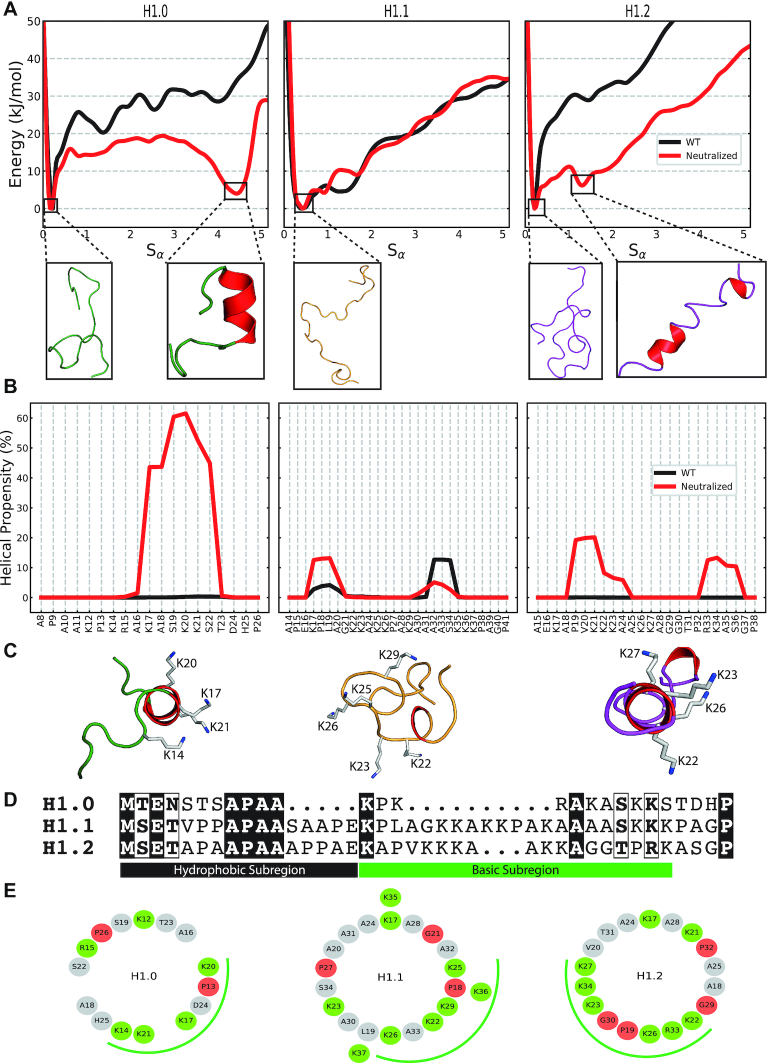
Inducible amphipathic helicities of the H1 NTDs. (**A**) The one-dimensional free energies along the Sα CV for the WT NTDs (black) and with neutralized Lys sidechains (red). The conformations at each of the free energy minima are illustrated with the α-helical motifs coloured in red. (**B**) The reweighted per-residue helical content from PTMetaD-WTE simulations of the WT NTDs (black) and with neutralized Lys sidechains (red). (**C**) Top-down illustrations of the induced NTD conformations from PTMetaD-WTE simulations with neutralized Lys sidechains. The α-helical motifs are coloured red and the residues within the positive ‘face’ are illustrated. (**D**) Sequence comparison of the of the hH1.0, hH1.1 and hH1.2 linker histone NTDs. (**E**) Helical wheel projections of the basic subregions of the three H1 NTDs. Basic Arg/Lys residues are in green while the helix-breaking Pro/Gly residues are in red. The basic ‘face’ of each subtype is illustrated with a green curve.

To further explore the structural heterogeneity of the NTDs, their size distributions were calculated and compared to the mean radius of gyration (*R*_g_) of a globular (77) and thermally denatured random-coil proteins (78). The structural heterogeneity of the NTDs is evident from the wide distributions of radius of gyration (Figure 1C). However, despite the large structural variation, most of the conformations adopted by the NTDs are compact, as shown by radii of gyration that are closer to the value predicted for a globular protein of similar length (Figure 1C and Materials and Methods). In contrast, Replica-Exchange with Solute Tempering (REST2) (88) simulations of the CTD of H1.0 (29) reveal extended conformations (Supplementary Figures S4 and S5). The differences in compaction between the two unstructured domains might plausibly be attributed to the uneven charge density within them. The 97-residue H1.0 CTD is predominantly composed of basic residues (42.2%, 41 Lys) while the 26-residue H1.0 NTD only has six basic residues (23.1%). To test this hypothesis, we split the H1.0 NTD into hydrophobic and basic subregions (Figure 2D) and recalculated the radius of gyration of each region from the T-REMD simulations (Supplementary Figure S6). Consistently, we observe that the basic subregion, with higher charge density, adopts extended conformations that yield the typical distribution of the radius of gyration of IDPs (Supplementary Figure S6). In contrast, the hydrophobic subregion adopts predominantly collapsed conformations that contribute to the comparatively compact NTD radius of gyration observed in Figure 1B.

This distinction between the two unstructured N- and C-terminal domains might provide a molecular explanation for their experimentally observed contrasting roles in mediating H1-nucleosome interactions (23,89). Extended states of the CTD might allow it to initiate the process of nucleosome recognition through long-range electrostatic interactions (89) while the collapsed conformations of the NTD might preclude this (23).

### H1 NTD undergoes a subtype-specific disorder-to-order transition upon charge neutralization

The NTD can be divided into two distinct subregions (90): the N-terminal subregion that lacks charged residues and is enriched in Ala, Pro and other non-aromatic hydrophobic residues and the basic subregion (close to the globular domain), which contains a high proportion of positive Lys/Arg residues crucial for electrostatic interactions with the DNA (Figure 2D) (90). Binding of H1 NTD to DNA is, hence, expected to significantly transform the structural behaviour of the second half of the NTD due to screening of the electrostatic repulsion among its numerous positively charged residues enabled by the interactions with the phosphate backbone (91).

We perform PTMetaD-WTE (68) simulations of the basic subregions of the three NTDs explored above in two conditions: (1) with standard charged Lys (WT), and (2) with neutralized Lys sidechains (neutralized; as an approximation to NTD charge neutralization by DNA). We use two collective variables (CV) that show complete diffusivity along the simulation (Supplementary Figure S7)—the radius of gyration (*S*_Rg_) and a function of the alpha-helical content (S_α_) (72)—and eight geometrically spaced temperature replicas from 300 to 450 K (see Materials and Methods) together with enhanced potential energy fluctuations (WTE - Supplementary Figure S1) (44). To measure alpha-helical content, *S*_α_ is defined as a function of the RMSD between the NTD and a six-residue ideal α-helix (72) (see Methods). We chose these parallel-tempering metadynamics method as it allows us to characterize the effects of charge neutralization in the statistical ensembles of the H1 NTDs and on their free energy surfaces as a function of their secondary structural content. In all cases, we run the simulations for 3.6 μs as we achieve convergence after 2.8 μs of accumulated sampling (350 ns per replica; see Supplementary Figure S8).

For the WT NTD systems, independently of the subtype, the free-energy profile along the *S*_α_ collective variable shows a single minimum at values of low helical content *S*_α_ ∼0.1, which evidences that the most energetically favourable conformations for all subtypes are those lacking secondary structure (Figure 2A). This is in agreement with the predominantly unstructured ensembles observed in our T-REMD simulations for the same systems (Figure 1). Upon charge neutralization, all three variants preserve a global minimum at *S*_α_ values of ∼0.1, signalling favourable unstructured conformations in all cases. However, interestingly, the neutralized NTDs of the H1.0 and H1.2 subtypes exhibit a second low energy minima at a higher value of *S*_α_ (Figure 2A), corresponding to conformations with helical secondary structural elements. Transient short-lived β-motifs were also observed, but with propensities that remain unchanged upon charge neutralization (Supplementary Figure S10). To discard the existence of other energy minima, the two-dimensional free energy profiles along the two CVs were plotted and compared between the two Lys charge states (see free energy surface (FES) plots in Supplementary Figure S9).

The minima of the H1.0 NTD at large *S*_α_ values (Sα=4.4) is nearly as stable as that of the unstructured conformations (Δ*E* = 3.6 kJ/mol). The helical propensity of the neutralized H1.0 NTD (Figure 2B), estimated using the reweighted probability distributions stemming from our PTMetaD-WTE simulations (see Materials and Methods) (79), shows that this is a single seven residue-long helix spanning the region _16_AKASKKS_22_. To validate this structural transition, we performed Biased-Exchange Metadynamics (BEMD) (Supplementary Discussion, Supplementary Table S2) of the whole length H1.0 NTD with neutralized Lys sidechains using varying force fields/water/ion parameters (Supplementary Table S3). While the extent of helicity varied, the span of the motif remained invariant throughout (Supplementary Figure S11).

Further inspection of this motif (Figure 2C), shows that forming a helix would concentrate the positively charged residues K14, K17, K20 and K21 on the same face of the helix, creating a charged face on the NTD that should favour interactions with DNA. Indeed, the projection of sidechains down the axis of an ideal helix (3.6 residues per turn, 100° between residues), known as helical wheel analysis, reveals that the sequence of amino acids in the H1.0 NTD is ideal for clustering charge along one face (Figure 2E), as occurs in many proteins that undergo a disordered-to-ordered transition upon DNA binding (92,93). Hence, once electrostatic repulsion is screened, the sequence of the H1.0 NTD leads to focussing of positive charge on one face of an α-helix favouring then the stability of H1.0 NTD-DNA contacts.

Although the H1.2 NTD with uncharged Lys sidechains also exhibits a second minimum corresponding to a partial helical conformation, this occurs at a much lower *S*_α_ value of 1.3 and with a free energy slightly higher (Δ*E* = 5.8 kJ/mol) (in-comparison to the unstructured conformation). In line with the lower *S*_α_ value, the per-residue helicity for this subtype shows two short and more transient helical motifs involving residues _19_PVKKKA_24_ and _33_RKAS_36_ (Figure 2B). Inspection of the H1.2 NTD sequence explains why this subtype is unable to form a single long helix: each of the two short helices starts with a ‘helix-initiating’ Pro and are separated by a helix-breaking double Gly (G29/G30) motif (94). This is consistent with a helix-Gly-Gly-helix motif observed in the mouse H1.4 NTD (>95% sequence similarity to hH1.2 NTD, Supplementary Figure S3) in the helix-stabilizing solvent TFE (87). Despite helicity in the H1.2 NTD case being shorter-lived than in the H1.0 subtype, transient partial folding and rigidification of the H1.2 NTD upon DNA binding would imply enabling a more compact configuration that concentrates positive charge (in this case three and two basic residues per helical motif) better than a random coil and favours stronger interactions with DNA.

In contrast to the two other H1 NTDs, the H1.1 subtype exhibits a one-dimensional free-energy surface along *S*_α_ that is invariant with the Lys charge state. The per-residue helicity plot in Figure 2B confirms only two small segments with low propensity (<10%).

The differences in folding propensities that we observe correlate with the percentage of positively charged residues and the lack of helix-breaking residues that each system has (see Table 1). That is, the H1.0 NTD subtype that possesses the highest folding propensity, has the highest concentration of positive residues and only one ‘helix-destabilizing’ residues. Consistently, the H1.1 NTD subtype that exhibits the weakest folding propensity, has the lowest positive charge concentration in the set and is interspersed with four ‘helix-destabilizing’ residues (95) (see Table 1 and Figure 2D). The helical-wheel analysis in Figure 2E further illustrates this trend with the H1.0 and H1.2 subtypes that display helical propensity also exhibiting significant built-in amphiphilicity and charge concentration within the basic ‘face’ (green curve) in contrast to the unstructured H1.1.

**Table 1.** Characteristic differences in the sequences of the NTD basic subregion of the H1.0, H1.1 and H1.2 subtypes. The basic subregion was considered as the segment between the first and last Arg/Lys residues within the NTD

Subtype	Length	No. of Arg/Lys	No. of Pro/Gly
H1.0	10	6 (60.0%)	1
H1.1	21	9 (42.8%)	3
H1.2	18	8 (44.4%)	4

### H1 NTD also undergoes a disorder-to-order transition upon DNA binding

**Figure 3.**
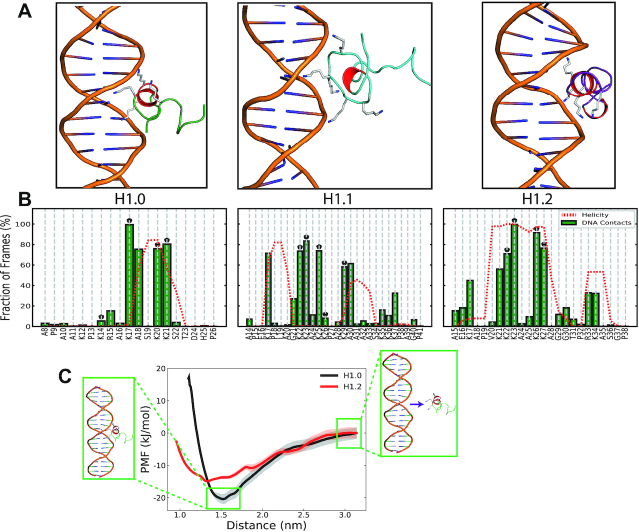
Interactions of the induced helical conformations with DNA. (**A**) Lowest energy docked conformations of the induced helical conformations with a linear 20-bp β-DNA strand. The α-helical motifs are in red and the Lys within the basic face are illustrated. (**B**) Interactions of specific residues within the NTD with DNA from the final 250 ns of simulations of the docked conformations. A contact was assumed if a non-hydrogen atom of the residue was within 3.2 Å of a non-hydrogen DNA atom. The average per-residue helicity during this final 250 ns is shown in red. The Lys residues within the basic face of each H1 NTD are marked with a black ‘*’. (**C**) PMF profiles for the binding of the H1.0 (black) and H1.2 (red) with the DNA major groove. The standard deviations of the calculated PMFs estimated form the bootstrapping are shown as shaded regions. Snapshots illustrate the initial and final window configurations for the H1.0 variant and the radial distance used as the CV (purple arrow). The energy profiles are shifted so that the energy at large distances is set to zero.

We now focus on determining if the charge-neutralization-induced helical conformations observed above are indeed reproducible when charge repulsion among Lys in the H1 NTD is instead screened via DNA binding. For this, we start by using HADDOCK (81) to rigid-body dock a NTD configuration representative of either the second minima (for the H1.0 and H1.2 subtypes) or the first minimum (for the H1.1 subtype) from our PTMetaD-WTE simulations (on the neutralized peptides) to a 20-bp ds-DNA segment (see Materials and Methods). Before the docking, we restore the full charges on the NTD Lys. In the case of H1.0 and H1.2, we consider the Lys residues within the amphipathic face (Figure 2C, E) as active residues for the docking interaction, in the case of H1.1 use K22, K23, K25, K26 and K27, as they are within a region with the highest charge density. Figure 3A shows the lowest energy HADDOCK cluster for the three subtypes. For the H1.0 and H1.2 subtypes, docking predicts the helix regions to fit snuggly within the DNA major groove (the four lowest energy clusters were similar for all three cases), facilitating interactions between the four Lys within the basic faces and DNA. Binding of helices to the major groove is amongst the most common DNA-protein interaction motifs with such interfaces present within helix-turn-helix proteins, zinc-binding proteins and leucine zipper proteins (96). In the case of H1.1 NTD docked configuration instead, there are only three basic residues (all within the unstructured region) that interact with DNA around the minor grove.

To assess the viability of the docked conformations and to investigate the implications of NTD differential folding across subtypes on DNA interactions, we run unbiased MD simulations of the DNA-docked NTD basic subregion systems with fully charged Lys sidechains. Our results confirm that charge-screening by DNA binding favours disorder-to-order transitions in all the H1 NTD subtypes studied. When interacting with the DNA strand, the induced helical configuration in the H1.0 NTD is stable across the 400 ns MD simulation (Supplementary Figure S12), with residues in the basic face (K17, K20, K21) of the helix accounting for the majority of interactions with the DNA (Figure 3B).

Simulations of the DNA-bound H1.2 NTD also show a prominent disorder-to-order transition upon DNA binding, with region _19_PVKKKAAKKAGG_30_ retaining the stable Lys-rich helix throughout the course of the simulation (see Supplementary Figure S12). Here, the four Lys K22, K23, K26 and K27 residues fall on the same side of the helix, making two contiguous helical turns that interact strongly with the DNA (Figure 3B), mainly at the major groove. A secondary small set of residues (_33_RKA_35_) with helical properties can also be observed. However, this secondary motif is too short to form a full helix and correlates with less prominent NTD-DNA interactions.

Contrary to the neutral-Lys PTMetaD-WTE simulations, the H1.1 NTD also forms two short helices (_17_KPLA_20_ and _29_KAAAA_33_) that are joined together by a Lys-rich unstructured loop (_21_GKKAKKPA_28_). Because the helices formed here are mainly devoid of charged residues, DNA interactions are instead mediated by the unstructured loop and the single charged residues at the edge of each helix. Enhanced fluidity of the binding region is consistent with the H1.1 NTD showing a reduced number of DNA-bound states (∼83% of the simulation frames) than the H1.0 and H1.2 cases (100%), where rather than focusing on the DNA major groove, the NTD promiscuously binds/unbinds the entire DNA strand (Supplementary Figure S13). Although the specific DNA interaction patterns of the disordered H1.1 segment depend on the initial configurations generated by HADDOCK's rigid body docking algorithm, our results (Figure 3B, Supplementary Figure S13) demonstrate a distinction between the promiscuous interactions of disordered segments and the stable interactions of structured segments.

To quantify the impact of this observed subtype-specific helical propensity on the protein's DNA binding ability, we took the two partially structured H1 NTD subtypes (i.e., H1.0 and H1.2) and, for each of them, independently simulated the binding process to a 20-bp ds-DNA strand by means of umbrella sampling simulations (see Methods). From the umbrella sampling simulations, we calculate the potential of mean force (PMF) as a function of the radial protein-DNA distance normal to the DNA’s helical axis (Figure 3C). The PMFs reveal that while the subtype with highest helical propensity, the H1.0 NTD, is stabilized by ∼20 kJ/mol (∼8*k*_B_*T*) upon DNA binding, the longer H1.2 NTD—with a decreased helical propensity—is stabilized by only ∼15 kJ/mol (∼6*k*_B_*T*) upon DNA binding. However, it should be noted that within the force fields, interactions between the oppositely charged amine and phosphate groups have not been explicitly calibrated (97). Thus, we repeated the PMF calculations with the Amberff14SB (98) and parmbsc1 (99) force fields together with the complete set of non-bonded fix (cufix) corrections (100). While the inclusion of the non-bonded amine–phosphate interaction corrections modified the free energy profile at short distances, the depth of the resulting well is equivalent (∼20 kJ/mol) (Supplementary Figure S14). These results suggest that a higher tendency of the H1 NTD subtypes to undergo a disorder-to-order transition (favoured by a shorter length and higher charge density) enhances their DNA binding propensity.

Finally, to further investigate the stability of the disorder-to-order NTD transition, we performed metadynamics simulations of the basic subregion of the H1.0 NTD (with normally charged Lys sidechains) in contact with a 20-bp ds-DNA strand, starting from an unstructured NTD configuration. We used two CVs: the alpha helicity (*S*_α_) and the number of electrostatic contacts (*S*_cont_) between the phosphate backbone and Lys/Arg sidechains (see Methods). Convergence was assessed using the time-evolution of the free-energy profiles (Supplementary Figure S15).

**Figure 4.**
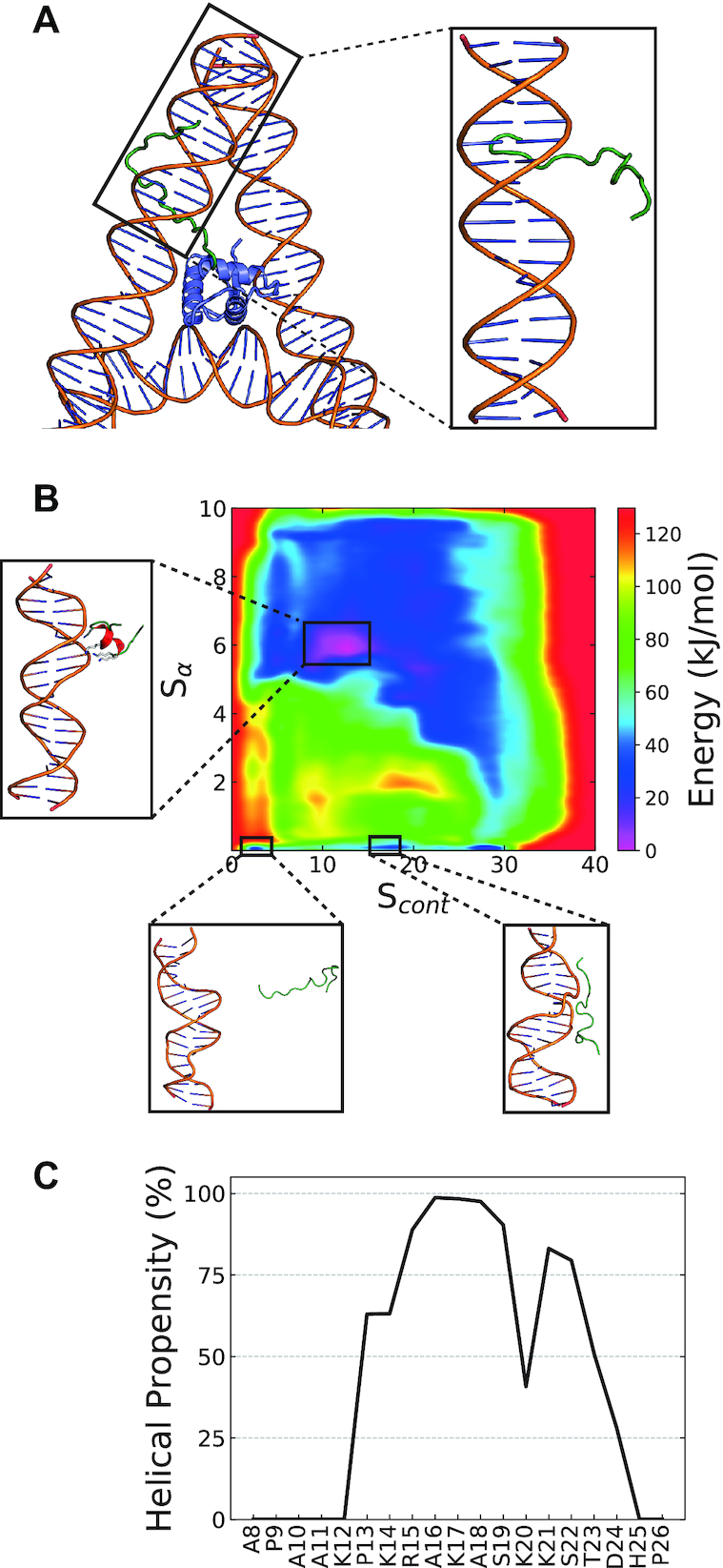
DNA induced secondary structure within the basic subregion of the H1.0 NTD. (**A**) Left: Constructing the initial configuration for metadynamics simulations – the NTD basic subregion (green) was built in an extended random coil configuration onto the on-dyad H1.0 globular domain (blue, PDB ID: 5NL0). Right: A 20-bp linker arm and the constructed NTD were then together considered the initial configuration. (**B**) Two-dimension free energy profiles of the NTD when interacting DNA along the two CVs – *S*_α_ and *S*_cont_. Three different configurations are illustrated: (1) the primary energy minima with a helical motif when bound to DNA, (2) unstructured and unbound and (3) DNA bound but unstructured. (**C**) The reweighted per-residue helical content of the H1.0 NTD basic subregion when interacting with DNA.

The simulations predict that upon DNA binding the NTD transitions from the unstructured configuration (*S*_α_ = 0, Figure 4) to a helical state; that is, the global minimum appears at *S*_α_ ∼5.8 and *S*_cont_ ∼12.5, which corresponds to the helical peptide bound to DNA (Figure 4B, Supplementary Figure S16). The resulting helix spans a larger region (_13_PKRAKASKKST_23_ in Figure 4C) than that predicted by our simulations with neutralized Lys sidechains and no DNA (_17_KASKKS_22_ in Figure 2B), suggesting that DNA binding effects are stronger than simple charge neutralization in triggering a disorder-to-order transition of the NTD. In addition, the interaction between the NTD and DNA emerges as non-specific with homogeneous contacts across the DNA strand (Supplementary Figure S17).

### Implications of NTD disorder-to-order transition on H1 nucleosome binding

After having determined that the NTDs of H1 undergo a subtype-specific disorder-to-order transition upon DNA binding, we explore the implications of this structural transition for H1-nucleosome binding. We first verify if a similar transition is observed when the H1 NTD forms part of a full H1 protein (with both globular and CTD domain) that is bound to a nucleosome. For this, we compare the behaviour of an isolated H1.0 NTD peptide to that of the NTD of H1 in our recent 5-μs long Biased-Exchange Metadynamics simulation of the full H1.0 bound to a 211-bp chromatosome (29) (SI Discussion, Supplementary Table S4). For a direct comparison to the nucleosomal results, we additionally repeated the ‘neutralized’ PTMetaD-WTE simulations with the full length H1.0 NTD (in-contrast to only the basic subregion discussed in the preceding sections).

**Figure 5.**
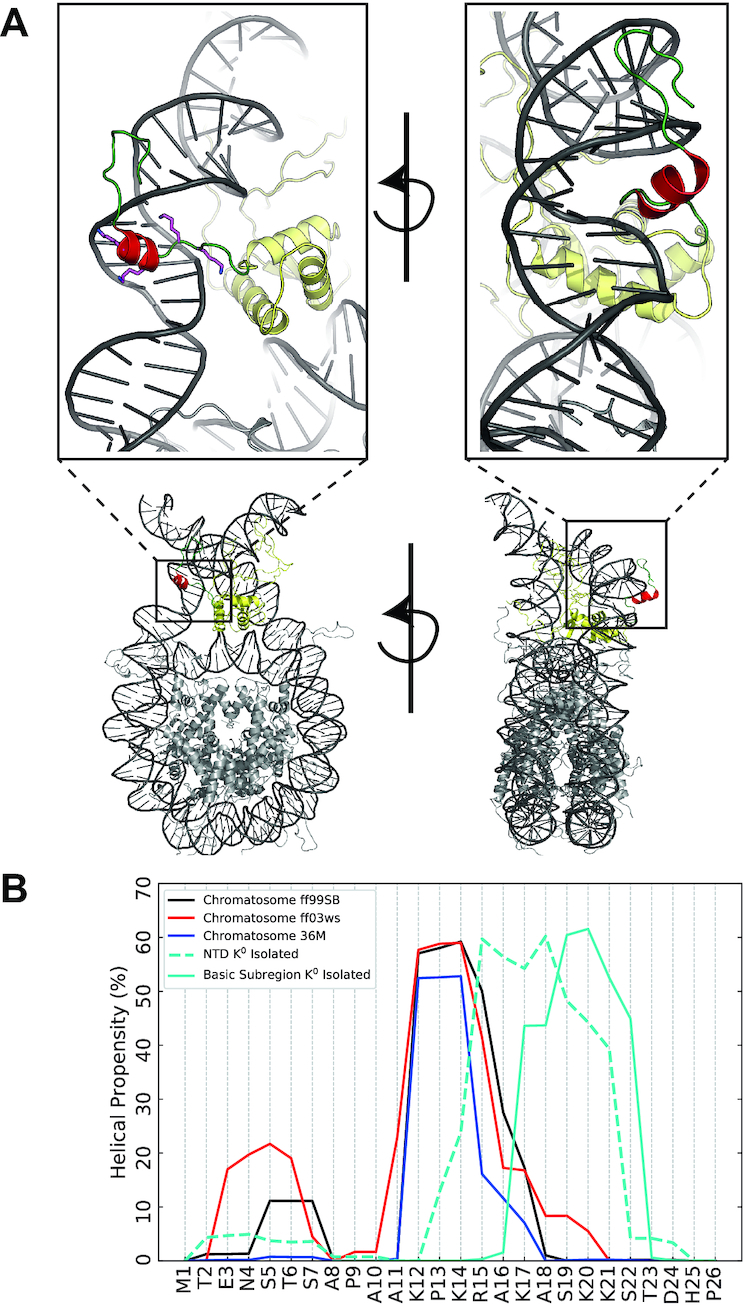
The N-terminal domain within the nucleosome. (**A**) Illustrations of the most-populated cluster of the NTD from 5-μs BEMD simulations of the full-length hH1.0 within a 211-bp nucleosome. The globular-domain is shown in yellow while the helical-motif within the NTD is in red. The four residues that constitute the basic ‘face’ from the helical-wheel analysis are illustrated. (**B**) Comparison of the per-residue helicity of the NTD within the nucleosome across different force fields (ff99SB-ildn, ff03ws, Charmm36M (SI Discussion)) and when isolated with neutralized Lys sidechains.

Figure 5B shows the per-residue structural propensity of the NTD within the nucleosome using multiple force-fields (Amber99SBildn, Amber ff03ws, Charmm36M (Supplementary Table S5)) and compares the results to those obtained from neutralized H1.0 segments. For all systems, the NTD displays a significant tendency to transition into a helical state. In comparison to the modelling of the shorter basic subregion (cyan, Figure 5B), modelling the entire NTD (dotted line, Figure 5B) allows the formation of a larger helical motif.

Within the nucleosome-bound systems, regardless of the force field, the helical motif is formed by the residues _12_KPKRAK_17_ and mediates interactions with DNA (Supplementary Figure S18). Upon nucleosome binding, the exact region where the helix forms is shifted slightly away from the point of attachment of the NTD to the globular domain, when compared to the helix in isolated NTD peptide. This difference may be plausibly attributed to the NTD within the nucleosome-bound system being subjected to additional configurational restraints imposed by its attachment to the globular domain of H1.

What is the meaning of these results for the binding of H1 to the nucleosome? Several different classifications for the relative nucleosomal binding affinities of the various H1 subtypes have been proposed (see Table 2). Despite variance across experimental methods, most studies have classified H1.0 as possessing the highest nucleosome binding affinity, followed by H1.2 and finally H1.1 (see Table 2). This trend is also consistent with H1.0 subtype overexpression being associated with quiescent chromatin/repressed gene expression (101), and the H1.2 and H1.1 subtypes localizing in transcriptionally active euchromatic regions (16). Remarkably, the observed binding affinities vary congruously with the ability of the different H1 NTD subtypes to form helical conformations upon charge-neutralization. Hence, it is plausible to propose that the NTD contributes to the differential nucleosomal binding of the subtypes through differences in its conformational preferences when within the nucleosome. That is, the higher nucleosome binding affinity of the H1.0 subtype might be in part due to the H1.0 NTD being able to form a stable amphipathic helix that increases the stability of the DNA-NTD complex, helping ‘glue’ the H1 to the nucleosome. Conversely, the longer and less-positive H1.1 NTD is disordered, which in-turn might result in weaker, fluctuating, and promiscuous interactions with the nucleosomal DNA (Figure 3B). Our simulations thus suggest a molecular mechanism contributing to explain the differential nucleosome binding affinities of the H1 subtypes.

**Table 2.** Summary of the experimental nucleosomal binding affinities of the linker histone subtypes. Experiment 5 only ranked the subtypes into two affinity categories. Experiments 6 and 7 only compared two specific subtypes and thus have no ‘intermediate’ ranking

ID	Low	Intermediate	High	Reference
1	H1.1, H1.10	H1.0, H1.2, H1.3	H1.4, H1.5	Clausell *et al.* (15)
2	H1.1	H1.2, H1.5	H1.0, H1.3, H1.4	Orrego *et al.* (104)
3	H1.1, H1.2	H1.3, H1.4, H1.5	H1.0	Flanagan *et al.* (23)
4	H1.1, H1.2	H1.0, H1.3	H1.4, H1.5	Th’ng *et al.* (16)
5	H1.2, H1.3	-	H1.4, H1.0	Oberg *et al.* (105)
6	H1.2	-	H1.0	George *et al.* (21)
7	H1.2	-	H1.0	Vyas *et al.* (32)

## CONCLUSIONS

We have used a combination of microsecond-long T-REMD, PTMetaD-WTE and BEMD simulations to sample the conformational landscape of the NTD of H1 subtypes H1.0, H1.1 and H1.2. When free in solution, our simulations predict that all three NTDs would predominantly adopt unstructured but partially collapsed conformations. Interestingly, when the Lys sidechains are neutralized either artificially by increasing the pH or physiologically by binding to DNA, the simulations show that reduced inter-sidechain repulsion is expected to be sufficient to induce a subtype-specific disorder-to-order transition. However, DNA-binding has a stronger effect than simple charge-reduction at triggering such transition. The ordered NTD structures in subtypes H1.0 and H1.2 are amphipathic helical conformations that orient the basic residues of the NTD along one ‘face’ of the helix, facilitating electrostatic interactions with the DNA major groove along that face which drives the peptide to a folded conformation acting as an ‘anchor’ between the DNA and H1 as previously hypothesized (102). By combining docking, unbiased MD simulations, and metadynamics simulations we show that the NTD conformational preferences translate into different patterns of DNA/NTD interactions and, hence, a differential ability of the different subtypes to mediate interactions of the H1 and the nucleosome.

Our simulations further reveal that the different charge density per unit length, the total length of the NTD, and interspersing of ‘helix-breaking’ Pro/Gly residues across subtypes, give rise to different degrees of helicity of the NTD; thereby, shorter charge-dense positive peptides devoid of Pro/Gly residues (H1.0 and H1.2) emerge as optimal for helical folding. Importantly, the varying degree of helicity upon DNA binding correlates with the experimentally observed binding affinities of the specific H1 subtypes, and the differences in the free energy stabilization of the various DNA-NTD complexes, with higher affinity subtypes exhibiting more prominent helical conformations. Specifically, upon charge neutralisation, the strongest binding H1.0 subtype forms a single contiguous helix spanning four turns, while the weakest binding H1.1 displays no helicity. This computationally predicted structural mechanism correlates with the experimentally observed effects of the H1.0/H1.2 NTD domain-swap that resulted in an exchange of their binding characteristics (32).

Our simulations postulate how subtle sequence differences in chromatin binding proteins can have a significant impact in their structural behaviour, and subsequently, their nucleosome binding affinity. Our results suggest a mechanism by which the structural behaviour of short charged-rich disordered domains of chromatin binding proteins could be crucially transformed after nucleosome binding. Prospectively, our observations can be exploited in the design of novel experiments to assess the structural preferences of the NTD of H1 in vitro and enhance our molecular understanding of the link between H1 NTD structure and the different roles of the various H1 subtypes in vivo (9). Our work also suggests that charge-reducing post-translational modifications like Lys acetylation (38,41) and Arg citrullination (103) can strongly impact the structural behaviour of the histone's terminal domains, especially those that are short and highly enriched in positively charged amino acids.

## DATA AVAILABILITY

The initial configurations, temperature distributions,docking results, PLUMED input files and GROMACS .mdp are available at the Cambridge Research repository (https://doi.org/10.17863/CAM.46225).

## Supplementary Material

gkaa285_Supplemental_File
