# Supplementary material for: Protein disorder-to-order transition enhances the nucleosome-binding affinity of H1
Source: Nucleic Acids Res. 2020 May 1;48(10):5318–31. doi: 10.1093/nar/gkaa285 (PMC7261198; doi:10.1093/nar/gkaa285)
Supplement: gkaa285_Supplemental_File [file gkaa285_supplemental_file.pdf]

Supplementary Information:

Protein disorder-to-order transition enhances the nucleosome-binding affinity of the H1 N-terminal domain

Akshay Sridhar[1], Modesto Orozco[2,3], Rosana Collepardo-Guevara[1,4,5*]

[1]Maxwell Centre, Cavendish Laboratory, University of Cambridge, JJ Thomson Avenue, CB3 0HE, Cambridge, United Kingdom
[2]Institute for Research in Biomedicine, Barcelona Institute of Science and Technology, Baldiri i Reixac, 19. 08028, Barcelona, Spain
[3]Department of Biochemistry and Biomedicine, University of Barcelona, Av. Diagonal 647. 08028, Barcelona, Spain
[4]Chemistry Department, University of Cambridge, Lensfield Road, CB2 1EW, Cambridge, United Kingdom
[5]Department of Genetics, University of Cambridge, CB2 3EH, Cambridge, United Kingdom

[*]To whom correspondence should be addressed
Email: rc597@cam.ac.uk

SUPPLEMENTARY DISCUSSION

Table S1: List, description and parameters of all the simulations performed in this work.

S. No.	System	Lysine State	Force-field	Water Model	Ion Parameters	Time	Simulation
1	H1.0 Entire NTD	Charged	ff99sb*-ildn-q (1, 2)	TIP3P (3)	NA - Aqvist (4)<br>CL – Dang (5)	56 replicas, 250 ns per replica	T-REMD
2	H1.0 Entire NTD	Charged	Charmm36M (6)	Modified TIP3P (6)	Beglov and Roux (7) with 'nbfix' (8, 9)	56 replicas, 250 ns per replica	T-REMD
3	H1.1 Entire NTD	Charged	ff99sb*-ildn-q	TIP3P	NA - Aqvist<br>CL – Dang	96 replicas, 250 ns per replica	T-REMD
4	H1.1 Entire NTD	Charged	Charmm36M	Modified TIP3P	Beglov and Roux with 'nbfix'	96 replicas, 250 ns per replica	T-REMD
5	H1.2 Entire NTD	Charged	ff99sb*-ildn-q	TIP3P	NA - Aqvist<br>CL – Dang	72 replicas, 250 ns per replica	T-REMD
6	H1.2 Entire NTD	Charged	Charmm36M	Modified TIP3P	Beglov and Roux with 'nbfix'	72 replicas, 250 ns per replica	T-REMD
7	H1.0 Basic Subregion	Charged	ff99sb*-ildn-q	TIP3P	NA - Aqvist<br>CL – Dang	8 replicas, 400 ns per replica	PT-MetaD WTE
8	H1.0 Basic Subregion	Neutral	ff99sb*-ildn-q	TIP3P	NA - Aqvist<br>CL – Dang	8 replicas, 400 ns per replica	PT-MetaD WTE
9	H1.0 Basic Subregion	Charged	ff99sb*-ildn-q	TIP3P	NA - Aqvist<br>CL – Dang	8 replicas, 400 ns per replica	PT-MetaD WTE
10	H1.0 Basic Subregion	Neutral	ff99sb*-ildn-q	TIP3P	NA - Aqvist<br>CL – Dang	8 replicas, 400 ns per replica	PT-MetaD WTE
11	H1.2 Basic Subregion	Charged	ff99sb*-ildn-q	TIP3P	NA - Aqvist<br>CL – Dang	8 replicas, 400 ns per replica	PT-MetaD WTE
12	H1.2 Basic Subregion	Neutral	ff99sb*-ildn-q	TIP3P	NA - Aqvist<br>CL – Dang	8 replicas, 400 ns per replica	PT-MetaD WTE
13	H1.0 Entire NTD	Neutral	ff99sb-ildn (10)	TIP3P	NA - Aqvist<br>CL – Dang	8 replicas, 400 ns per replica	PT-MetaD WTE
14	H1.0 Entire NTD	Neutral	ff03ws (11)	TIP4P-2005 (12)	NA - Aqvist<br>CL – Dang	4 replicas, 200 ns per replica	BEMD
15	H1.0 Entire NTD	Neutral	ff99sb*-ildn-q	TIP3P	Joung and Cheatham (13)	4 replicas, 200 ns per replica	BEMD
16	H1.0 Basic Subregion + ds-DNA	Charged	Charmm36M + Charmm DNA	Modified TIP3P	Beglov and Roux with 'nbfix'	600 ns	Metadynamics
17	H1.0 Basic Subregion + ds-DNA	Charged	ff99sb*-ildn-q + parmbsc0	TIP3P	NA - Aqvist<br>CL – Dang	600 ns	Metadynamics
18	Docked H1.0 Basic Subregion + ds-DNA	Charged	Charmm36M + Charmm DNA (14)	Modified TIP3P	Beglov and Roux with 'nbfix'	400 ns	Unbiased MD
19	Docked H1.1 Basic Subregion + ds-DNA	Charged	Charmm36M + Charmm DNA	Modified TIP3P	Beglov and Roux with 'nbfix'	400 ns	Unbiased MD
20	Docked H1.2 Basic Subregion + ds-DNA	Charged	Charmm36M + Charmm DNA	Modified TIP3P	Beglov and Roux with 'nbfix'	400 ns	Unbiased MD
21	Docked H1.0 Basic Subregion + ds-DNA	Charged	ff99sb*-ildn-q + parmbsc0 (15)	TIP3P	NA - Aqvist<br>CL – Dang	400 ns	Unbiased MD
22	Docked H1.1 Basic Subregion + ds-DNA	Charged	ff99sb*-ildn-q + parmbsc0	TIP3P	NA - Aqvist<br>CL – Dang	400 ns	Unbiased MD

23	Docked H1.2 Basic Subregion + ds-DNA	Charged	ff99sb*-ildn-q + parmbsc0	TIP3P	NA - Aqvist CL – Dang	400 ns	Unbiased MD
24	H1.0 Basic Subregion + ds-DNA	Charged	Charmm36M + Charmm DNA	Modified TIP3P	Beglov and Roux with 'nbfix'	20 windows, 100 ns	Umbrella Sampling
25	H1.2 Basic Subregion + ds-DNA	Charged	Charmm36M + Charmm DNA	Modified TIP3P	Beglov and Roux with 'nbfix'	20 windows, 100 ns	Umbrella Sampling
26	H1.0 Basic Subregion + ds-DNA	Charged	Amberff14SB (16) + parmbsc1 (17) + cufix (18, 19)	TIP3P	Joung and Cheatham	20 windows, 100 ns	Umbrella Sampling

Description of additional simulations

Biased Exchange Metadynamics of Isolated N-terminal Domains

The conformational ensembles adopted by Intrinsically Disordered Proteins (IDP) are dependent on the choice of force fields (20), water models (21), ion parameters (22) and Collective Variables (23). Here, a set of additional simulations were thus performed to validate the PTMetad-WTE results that predicted a charge neutralization induced transition to a helical conformation.

These simulations of the N-terminal domain's basic subregion with neutralized sidechains utilized the Well-Tempered (24) Biased-Exchanged (25) Metadynamics (26) (BEMetaD) method. Here, multiple replicas of {i} of the system are simulated at the same temperature while being based along different Collective Variables (S_i). At regular intervals, the atomic co-ordinates of two walkers 'a' and 'b' are exchanged with the probability

$$P = min\left\{1, exp\left(\frac{1}{k_B T}\left[V_G^a(x^a) + V_G^b(x^b) - V_G^a(x^b) - V_G^b(x^a)\right]\right)\right\}$$

where V_G^i is the history-dependant metadynamics potential along CV *'i'* and x^i are the atomic coordinates of replica *'i'. The* exchanges thus allow each walker to independently diffuse along each CV space unencumbered by minima along correlated CVs. The BEMetaD simulations used four walkers (Table S1) and replica '1' was unbiased ($V_G^1 = 0$).

Table S2: The Metadynamics biasing CVs within the validation BEMetaD simulations.

Replica Number	CV
1	None (Unbiased)
2	S_α
3	S_β
4	S_{rg}

The replica '3' biases the β-sheet content within the IDP (27) and is defined as

$$S_\beta = \sum \frac{1 - \left(\frac{\Delta RMSD}{R_0}\right)^n}{1 - \left(\frac{\Delta RMSD}{R_0}\right)^m}$$

where R_0, n and m are 0.08 nm, 8 and 12 respectively. ΔRMSD is the root mean square difference of six residue segments between the configuration and ideal β-conformations.

The validation BEMetaD simulations with neutralized sidechains were performed on the basic subregion of the NTD with the most helical content – H1.0. These simulations used two different combination of force fields, ion and water parameters (Table S3).

Table S3: Parameters utilized in the BEMetaD simulations.

S. No.	Force Field	Water Model	Ion Parameters
1	Amber ff03ws (11)	TIP4P-2005 (12)	NA - Aqvist CL – Dang
2	Amber ff99sb*-ildn-q (1, 2)	TIP3P (3)	Joung and Cheatham (13)

To generate initial configurations, the NTDs were built in extended configurations using the *tleap* module of Amber (28) and subsequently condensed using 5 ns of simulations in implicit solvent. The BEMetaD simulations were performed for 200 ns per replica to generate an accumulated sampling of 800 ns. Metadynamics biasing gaussians were added every 500 trajectory steps and exchanges between replicas were attempted every 10 ps. The trajectories were reweighted using the methodology of Bonomi et al. (29) and secondary structures were assigned using DSSP (30). The secondary structural propensities from each parameter set and the comparison to the PTMetaD-WTE simulations are plotted in Figure S10.

Biased Exchange Metadynamics of whole length H1.0 within the Nucleosome

The specifics of the whole length H1.0 simulations within the nucleosome are detailed in our work (31) and briefly summarized here.

Model Building: The atomic coordinates for the 211-bp chromatosome with two symmetric 32-bp DNA linker arms were initially extracted from one of the central nucleosomes in the 1ZBB tetra-nucleosome structure (32). The eight histone proteins were then replaced by those from the 1.9 Å resolution 1KX5 structure (33) containing histone tails. The human H1.0 sequence was obtained from the Uniprot Consoritum (34) (ID: P07305) and Modeller (35) was used to create a homology model of the GD of H1.0 with H5 (PDB 4QLC) (36) as the template. This modelled H1.0 GD was positioned on the nucleosome by overlaying it to H5 in the 4QLC structure. The unstructured NTD and CTD of H1.0 were independently built in extended random coil configuration using VMD (37). A standard REMD simulation of this CTD was performed and a random configuration capable of fitting between the linker arms was selected. The NTD and chosen CTD were attached to the previously built H1.0 model using Pymol (38). Finally, a short 1 ns MD simulation of this conformation was performed in the GBSA implicit solvent (39, 40) to condense the terminal domains. This condensed system was then solvated in an octahedral box for the simulations.

BEMD Simulations: Five different replicas were used (one unbiased replica and four metadynamics-biased replicas) and exchanges were attempted between them at fixed intervals. Table S4 below summarizes the CVs applied to the CTD along each of the five replicas. **However, the NTD was unbiased in all replicas**. Three different force field combinations were used and summarized in Table S5. Metadynamics biasing gaussians were added every 500 trajectory steps and exchanges between replicas were attempted every 10 ps.

Table S4: CV applied to the H1.0 CTD in the BEMetaD simulations of the whole nucleosome (31).

Replica Number	CV
1	None (Unbiased)
2	S_α
3	S_β
4	S_{cont}
5	S_{rg}

Table S5: Force field parameters utilized in the BEMetaD simulations of the whole nucleosome (31).

S. No.	Force Field	Ion Parameters	Time
1	Amber99sb-ILDN + parmbsc0 + TIP3P	Aqvist and Dang	5 replicas, 1μs per replica
2	Amber03ws + parmbsc0 + TIP4P-2005	Aqvist and Dang	5 replicas, 750 ns per replica
3	Charmm36M + Charmm36 DNA + Modified TIP3P	Beglov and Roux with 'nbfix'	5 replicas, 500 ns per replica

SUPPLEMENTARY FIGURES

SI Figure 1

Energy distribution of replicas at 8 geometrically spaced temperature intervals between 300K and 450K. **(Left)** In the standard Parallel tempering and **(Right)** In the Well-Tempered Ensemble.

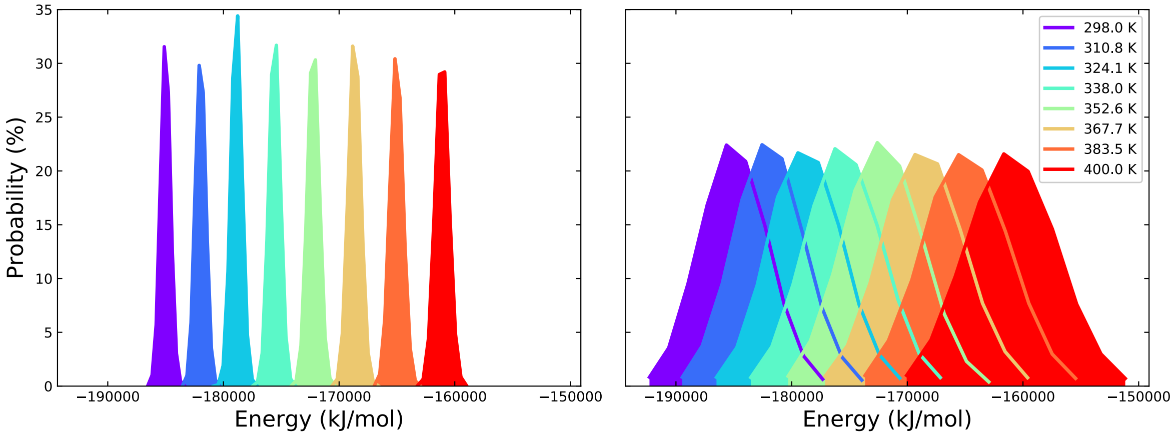

SI Figure 2

Secondary structural propensities from the final 250 ns of REMD simulations of the hH1.0, hH1.1 and hH1.2 N-terminal domains using the Charmm36M (6) forcefield.

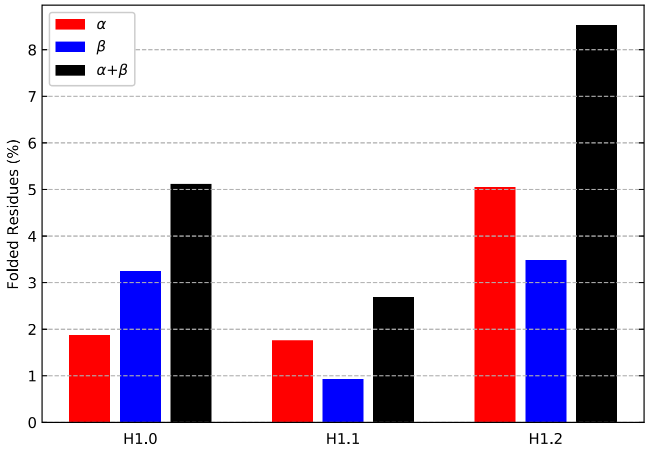

SI Figure 3

Sequence comparison between the two LH NTD orthologs predicted to exist in a double helical conformation - hH1.2 in this work and mH1.4 from the work of Vila et al. (41).

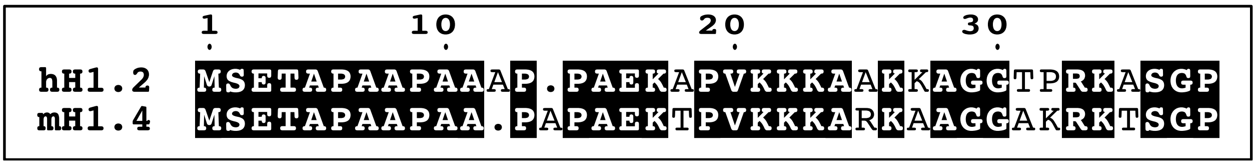

SI Figure 4

Distribution of the Radius of Gyration (Rg) of the H1.0 CTD Cα atoms from Replica-Exchange with Solute Tempering (REST2) simulations (42) of the domain in explicit solvent and 0.15 M Na/Cl. The theoretically predicted regressions for random-coil and globular proteins of similar length (43, 44) are shown in red/blue respectively.

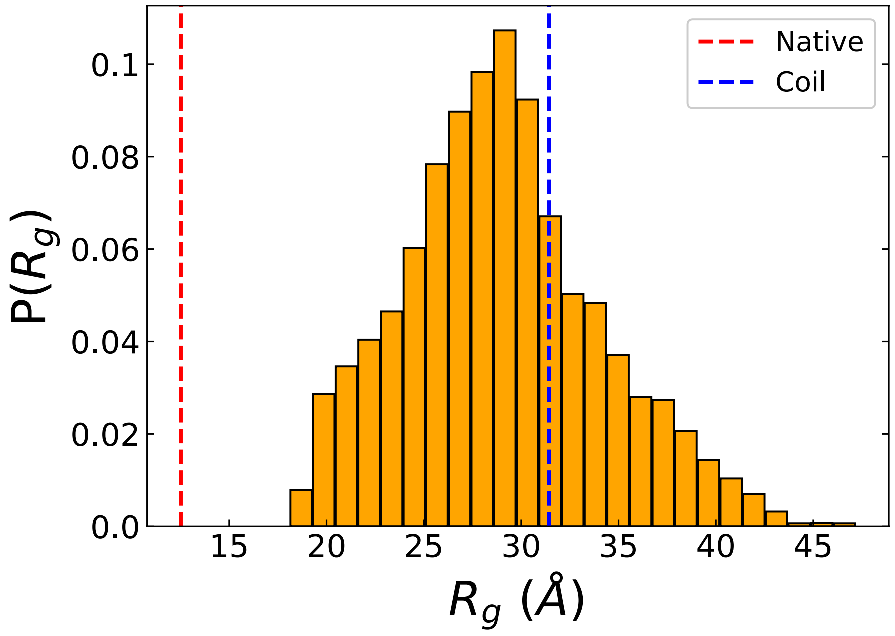

SI Figure 5

Distributions of solvent accessible surface area (SASA) of the N- and C-terminal domains calculated using a probe radius of 0.14 nm. The NTD SASA was calculated from the lowest temperature trajectory of the T-REMD simulations. For the CTD, the SASA was calculated from REST2 simulations of the domain in explicit solvent and 0.15 M Na/Cl. To allow comparison between the domains with different sizes, the CTD SASA was calculated and averaged for three segments (98-123, 124-149, 150-175) with a length similar to that of the NTD – 26.

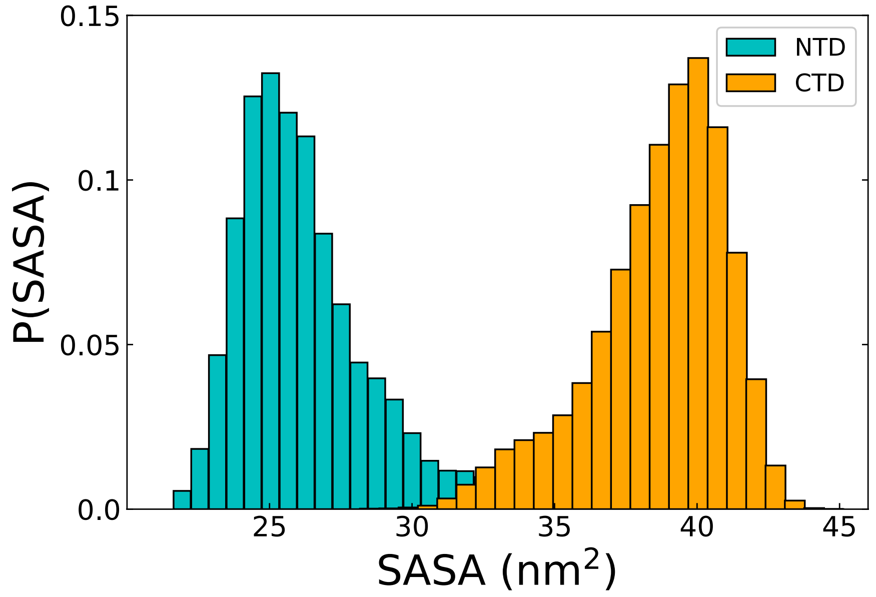

SI Figure 6

Distribution of the Radius of Gyration (Rg) of the H1.0 NTD hydrophobic (Top) and basic (Bottom) subregions from Temperature Replica Exchange (T-REMD) simulations. The theoretically predicted regressions for random-coil and globular proteins of similar length (43, 44) are shown in red/blue respectively.

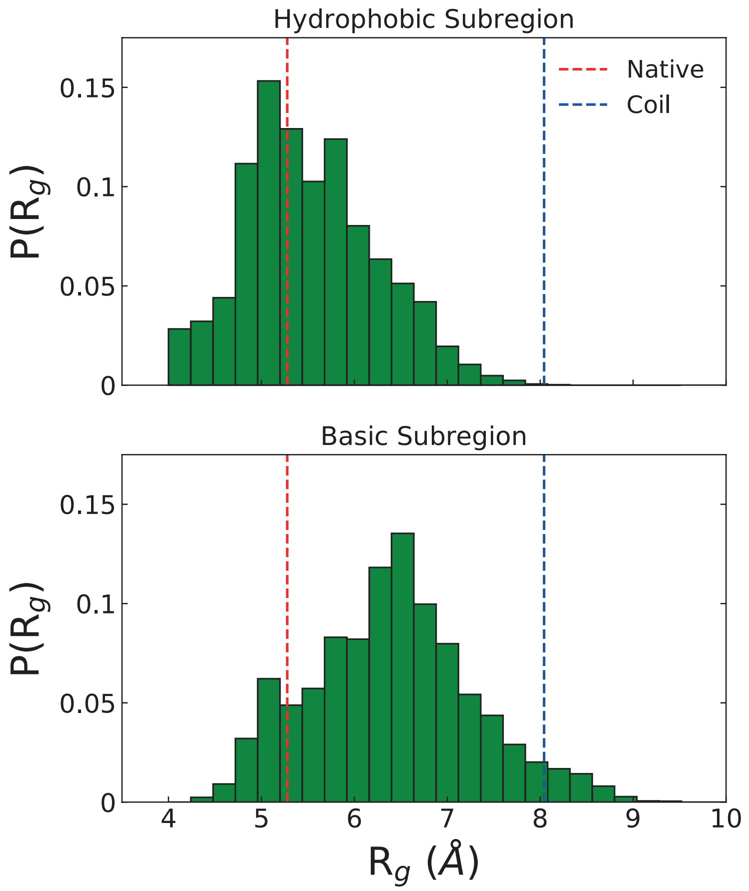

SI Figure 7

Convergence of the PT-WTE Metadynamics simulations assessed by the free diffusivity of the N-terminal domain IDP along the two Collective Variables S_α (27) and R_g.

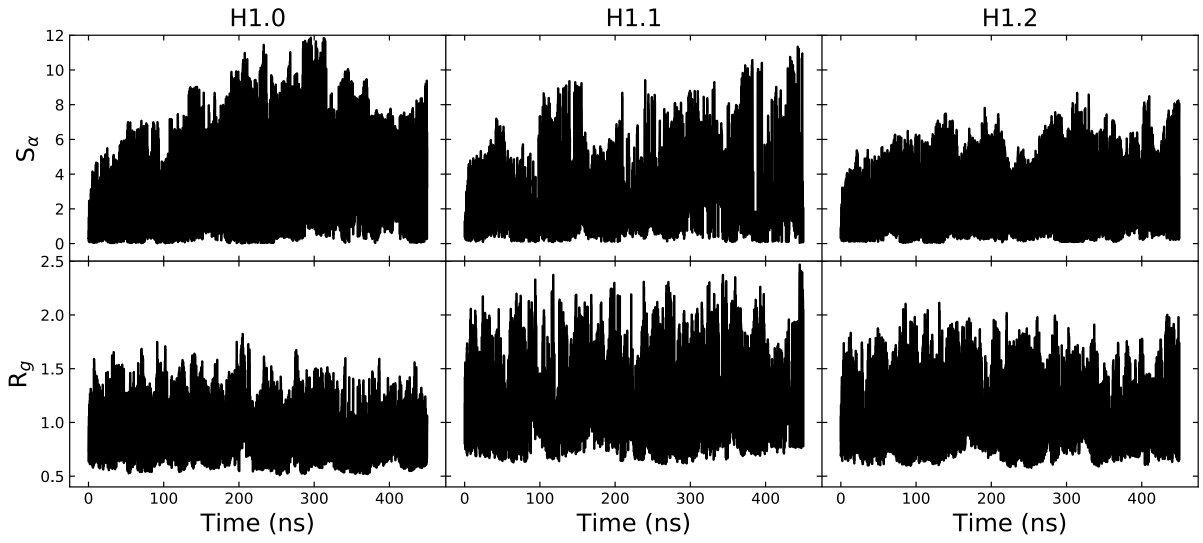

SI Figure 8

Convergence of the PT-WTE Metadynamics simulations assessed by the unsigned free energy difference between the two deepest energy minima as a function of time.

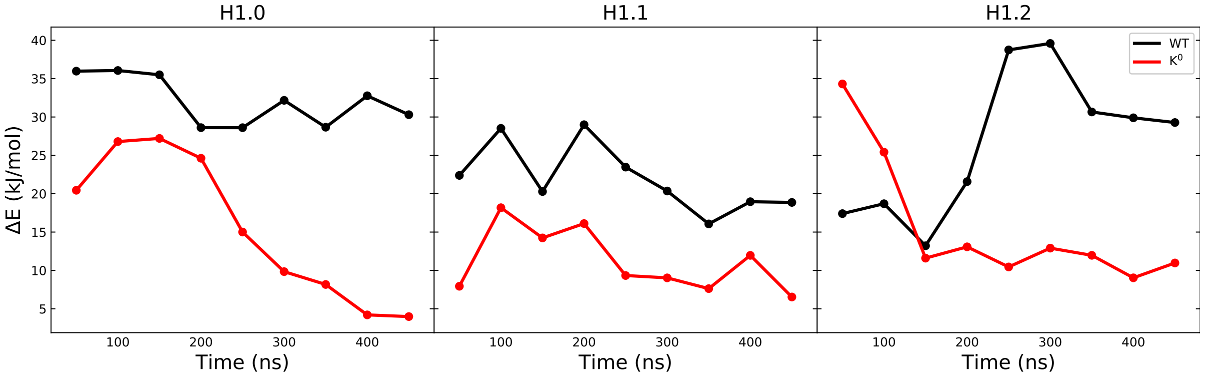

SI Figure 9

Free energy surfaces at 300 K of the NTD basic subregions along the two CVs – S_α and R_g obtained from the PT-WTE Metadynamics simulations. The energy basins 'induced' when the Lysine side-chains are uncharged are highlighted.

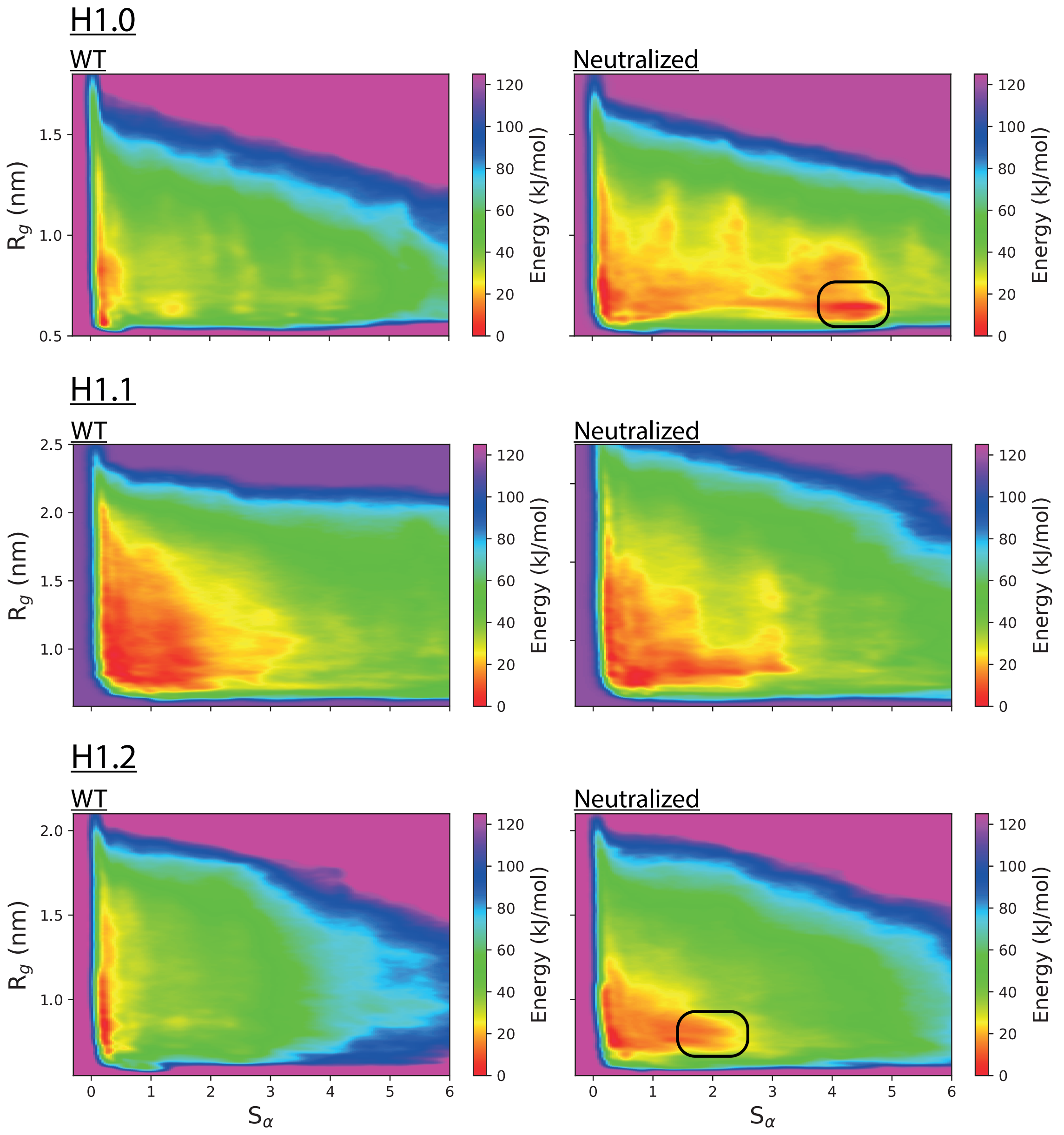

SI Figure 10

The per-residue β-motif propensity of the three Linker histone N-terminal domains from the PT-WTE Metadynamics simulations that is relatively invariant with the Lysine charge states.

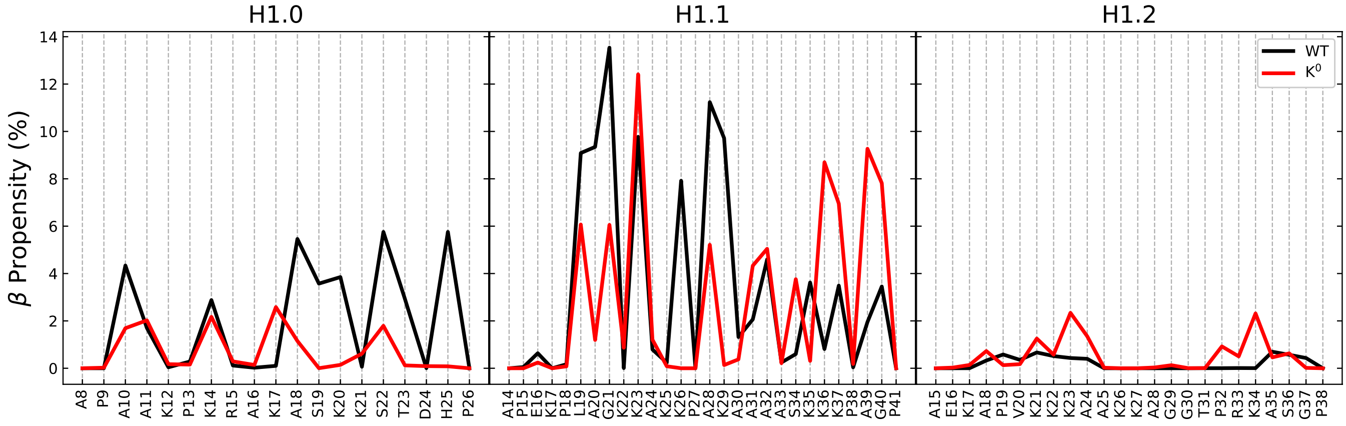

SI Figure 11

The per-residue helical propensity of the H1.0 NTD from BEMD simulations (with neutralized Lys) of the whole NTD segment using varying force field parameters (Blue/Red, SI Table S3). The per-residue helicity from PTMetad-WTE simulations (with neutralized Lys) of whole length H1.0 NTD (Black) and basic-subregion (cyan) are also plotted.

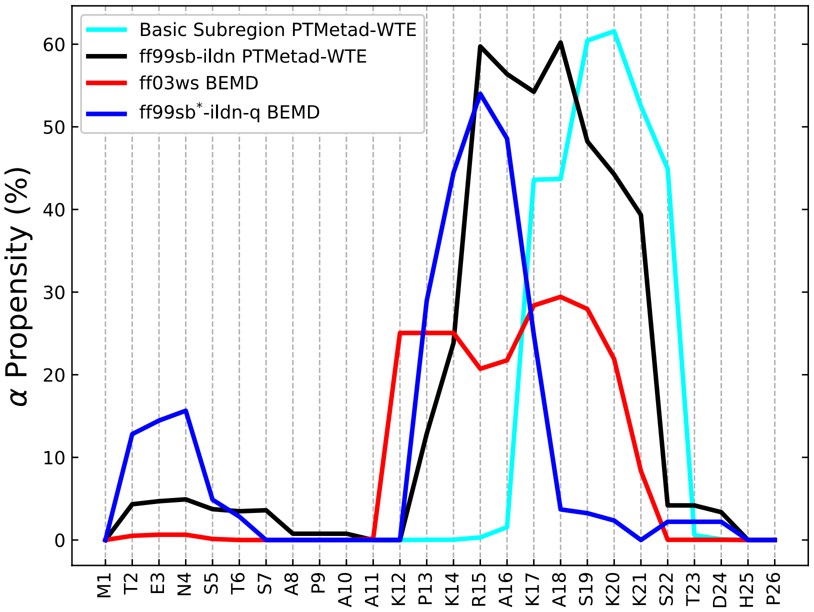

SI Figure 12

Per-frame secondary structure of the H1.0, H1.1 and H1.2 NTD basic sub-region IDRs over the course of an unbiased MD simulation when initiated from the HADDOCK (45) docking conformation. A simplified scheme is used where a 3_{10}- and alpha-helix are both considered an α-motif. Secondary structures were assigned using DSSP (30) within the MDTraj (46) library.

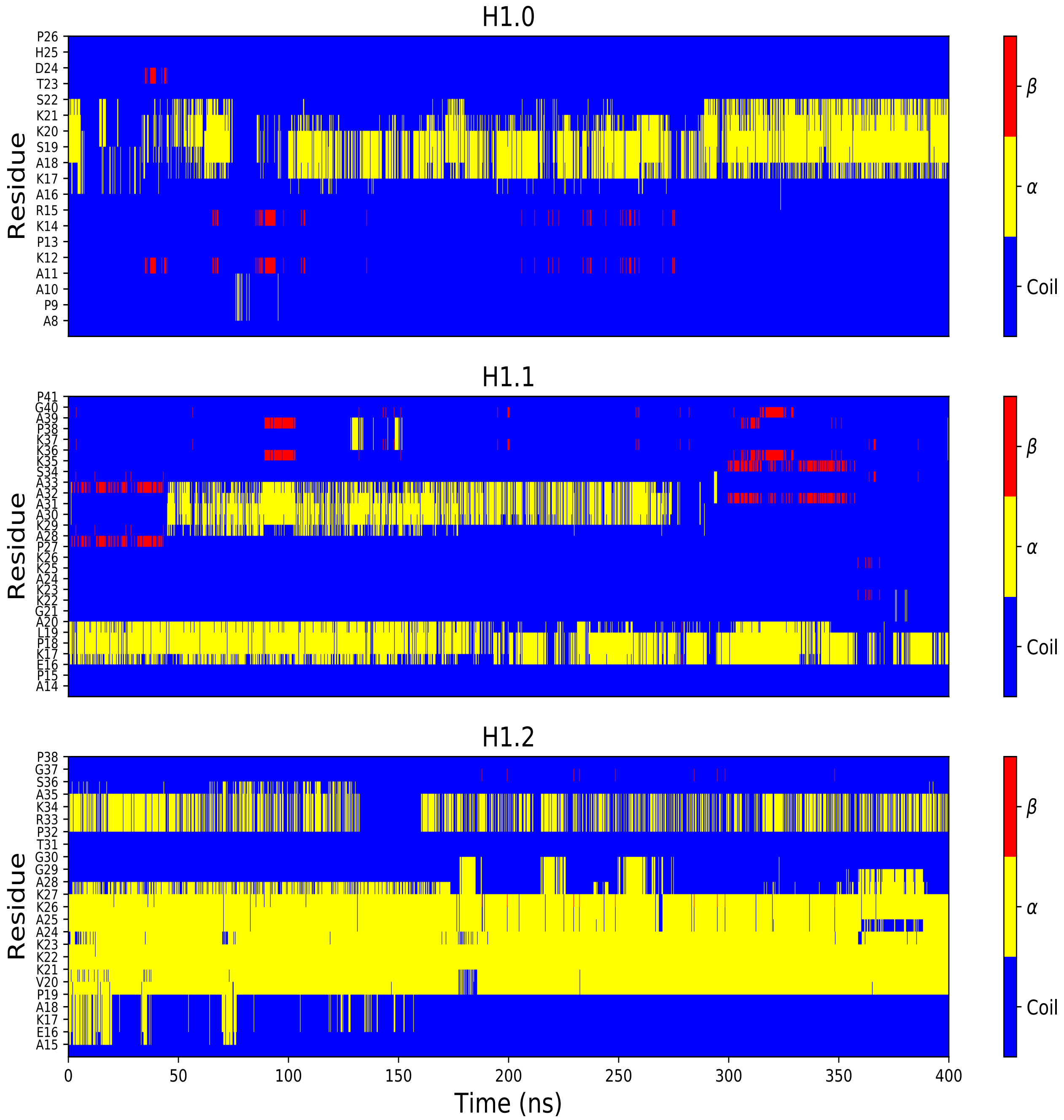

SI Figure 13

Normalized number of interactions per base-pair of the DNA strand with the three LH subtypes from the unbiased MD simulations. A contact is assumed if a non-hydrogen atom of the base-pair is within 3.2 Å of a non-hydrogen atom of the Linker Histone.

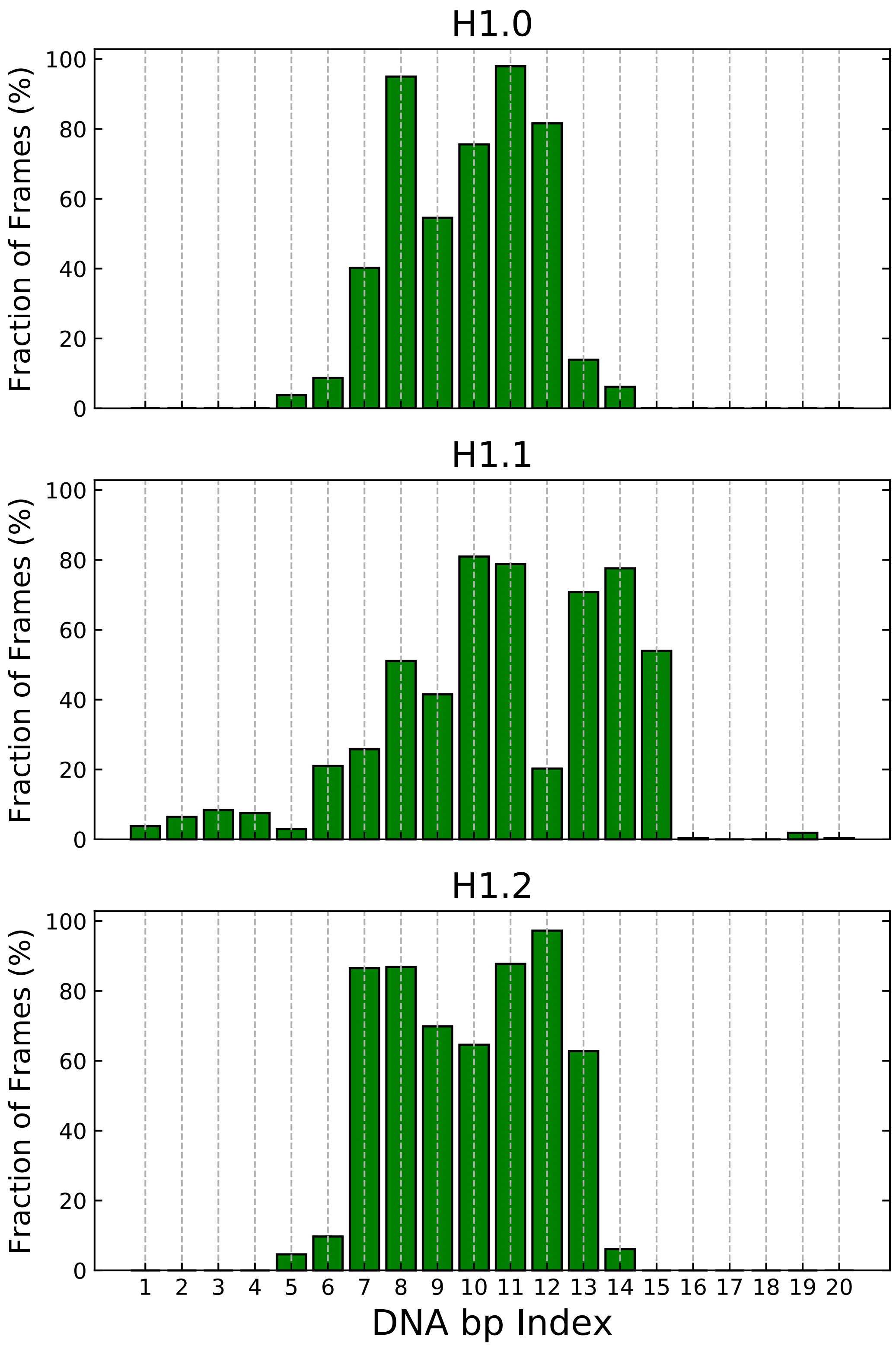

SI Figure 14

PMF profiles for the binding of the H1.0 basic subregions to the DNA major groove using the Charmm36M/Charmm36DNA (black) and Amberff14SB/parmbsc1 (red) forcefields. The standard deviations of the calculated PMFs estimated from the bootstrapping are shown as shaded regions. The energy profiles are shifted to set the energy at large distances to zero.

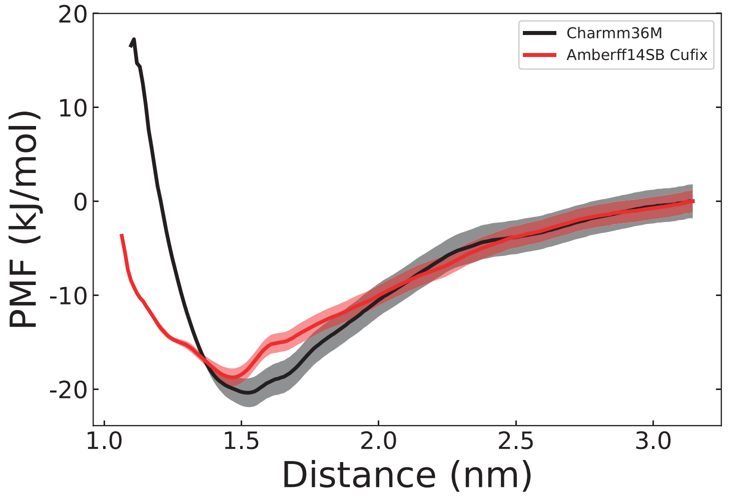

SI Figure 15

Convergence of the H1.0 NTD-DNA metadynamics simulations assessed by plotting **(A)** the unsigned free energy difference between the two deepest energy minima as a function of time and **(B)** the evolution of the free energy profile along the Sα CV as a function of time.

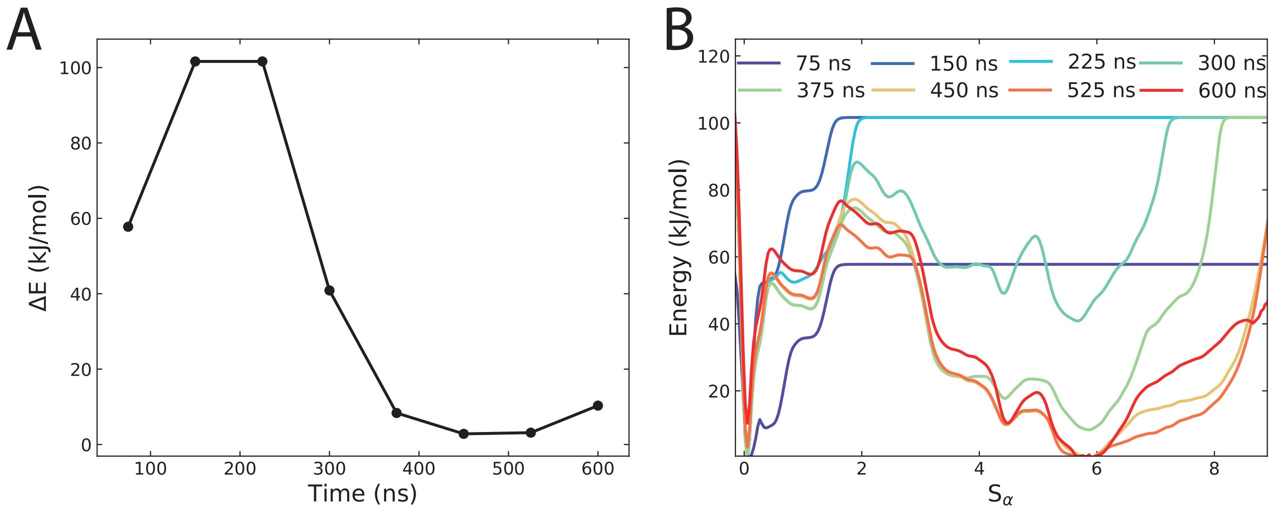

SI Figure 16

One-dimensional free energy along the Sα CV of the H1.0 NTD basic subregion when interacting with DNA calculated from metadynamics simulations (red). For comparison, the free energy profile of the basic subregion when isolated calculated from PTMetad-WTE simulations is shown (black).

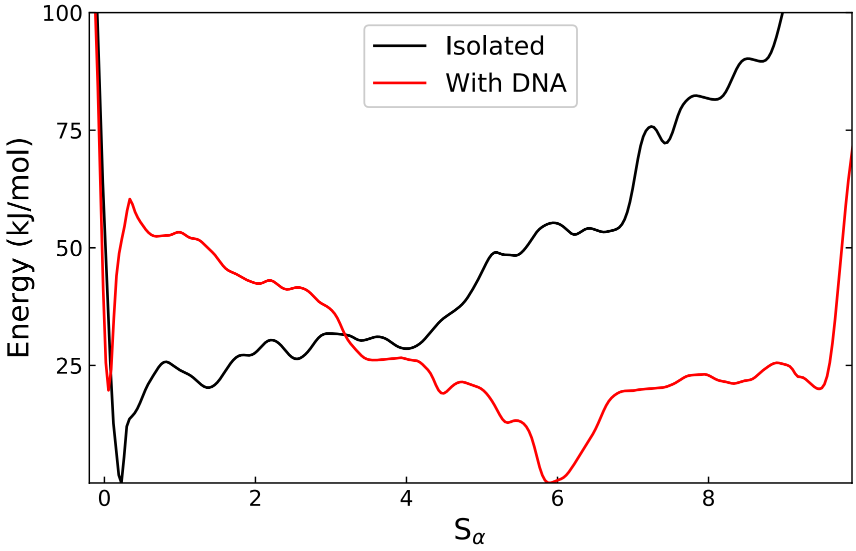

SI Figure 17

Normalized number of interactions per base-pair of the DNA strand from Metadynamics simulations of the H1.0 NTD basic subregion with DNA. A contact is assumed if a non-hydrogen atom of the base-pair is within 3.2 Å of a non-hydrogen atom of the NTD.

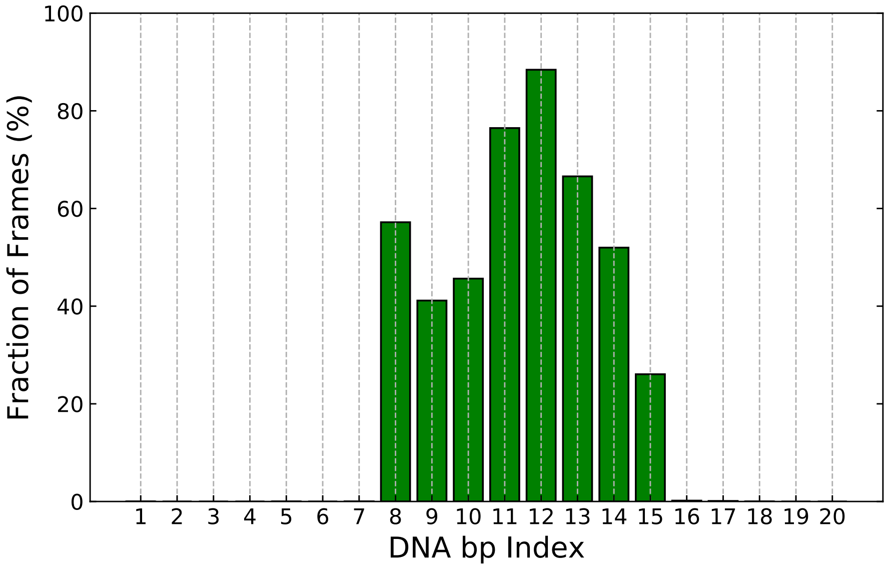

SI Figure 18

The normalized per-residue interactions of the NTD from BEMD simulations (31) of the full-length H1.0 within the nucleosome (ff99sb-ildn (10) for proteins and parmbsc0 (15) for DNA). A contact was assumed a non-hydrogen atom of the residue was within 3.2 Å of a non-hydrogen DNA atom. The residues within the basic face from helical wheel analysis are marked with a red 'o'.

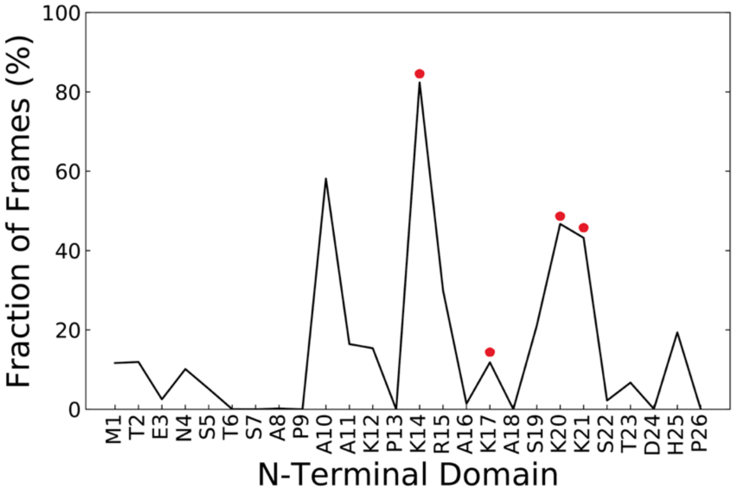

SI References

1. Best,R.B. and Hummer,G. (2009) Optimized molecular dynamics force fields applied to the helix-coil transition of polypeptides. *Journal of Physical Chemistry B*, **113**, 9004–9015.
2. Best,R.B., De Sancho,D. and Mittal,J. (2012) Residue-specific α-helix propensities from molecular simulation. *Biophysical Journal*, **102**, 1462–1467.
3. Jorgensen,W.L., Chandrasekhar,J., Madura,J.D., Impey,R.W. and Klein,M.L. (1983) Comparison of simple potential functions for simulating liquid water. *Journal of Chemical Physics*, **79**, 926–935.
4. Åqvist,J. (1990) Ion-water interaction potentials derived from free energy perturbation simulations. *Journal of Physical Chemistry*, **94**, 8021–8024.
5. Dang,L.X. (1995) Mechanism and Thermodynamics of Ion Selectivity in Aqueous Solutions of 18-Crown-6 Ether: A Molecular Dynamics Study. *Journal of the American Chemical Society*, **117**, 6954–6960.
6. Huang,J., Rauscher,S., Nawrocki,G., Ran,T., Feig,M., De Groot,B.L., Grubmüller,H. and MacKerell,A.D. (2016) CHARMM36m: An improved force field for folded and intrinsically disordered proteins. *Nature Methods*, **14**, 71–73.
7. Beglov,D. and Roux,B. (1994) Finite representation of an infinite bulk system: Solvent boundary potential for computer simulations. *The Journal of Chemical Physics*, **100**, 9050–9063.
8. Luo,Y. and Roux,B. (2010) Simulation of osmotic pressure in concentrated aqueous salt solutions. *Journal of Physical Chemistry Letters*, **1**, 183–189.
9. Venable,R.M., Luo,Y., Gawrisch,K., Roux,B. and Pastor,R.W. (2013) Simulations of anionic lipid membranes: Development of interaction-specific ion parameters and validation using NMR data. *Journal of Physical Chemistry B*, **117**, 10183–10192.
10. Lindorff-Larsen,K., Piana,S., Palmo,K., Maragakis,P., Klepeis,J.L., Dror,R.O. and Shaw,D.E. (2010) Improved side-chain torsion potentials for the Amber ff99SB protein force field. *Proteins: Structure, Function and Bioinformatics*, **78**, 1950–1958.
11. Best,R.B., Zheng,W. and Mittal,J. (2014) Balanced protein-water interactions improve properties of disordered proteins and non-specific protein association. *Journal of Chemical Theory and Computation*, **10**, 5113–5124.
12. Abascal,J.L.F. and Vega,C. (2005) A general purpose model for the condensed phases of water: TIP4P/2005. *Journal of Chemical Physics*, **123**.
13. Joung,I.S. and Cheatham,T.E. (2008) Determination of Alkali and Halide Monovalent Ion Parameters for Use in Explicitly Solvated Biomolecular Simulations. *Journal of Physical Chemistry B*, **112**, 9020–9041.
14. Hart,K., Foloppe,N., Baker,C.M., Denning,E.J., Nilsson,L. and MacKerell,A.D. (2012) Optimization of the CHARMM additive force field for DNA: Improved treatment of the BI/BII conformational equilibrium. *Journal of Chemical Theory and Computation*, **8**, 348–362.
15. Pérez,A., Marchán,I., Svozil,D., Sponer,J., Cheatham,T.E., Laughton,C.A. and Orozco,M. (2007) Refinement of the AMBER force field for nucleic acids: Improving the description of α/γ conformers. *Biophysical Journal*, **92**, 3817–3829.
16. Maier,J.A., Martinez,C., Kasavajhala,K., Wickstrom,L., Hauser,K.E. and Simmerling,C. (2015) ff14SB: Improving the Accuracy of Protein Side Chain and Backbone Parameters from ff99SB. *Journal of Chemical Theory and Computation*, **11**, 3696–3713.
17. Ivani,I., Dans,P.D., Noy,A., Pérez,A., Faustino,I., Hospital,A., Walther,J., Andrio,P., Goñi,R., Balaceanu,A., *et al.* (2015) Parmbsc1: a refined force field for DNA simulations. *Nature Methods*, **13**, 55–58.
18. Yoo,J. and Aksimentiev,A. (2016) Improved Parameterization of Amine-Carboxylate and Amine-Phosphate Interactions for Molecular Dynamics Simulations Using the CHARMM and AMBER Force Fields. *Journal of Chemical Theory and Computation*, **12**, 430–443.
19. Yoo,J. and Aksimentiev,A. (2018) New tricks for old dogs: Improving the accuracy of biomolecular force fields by pair-specific corrections to non-bonded interactions. *Physical Chemistry Chemical Physics*, **20**, 8432–8449.
20. Robustelli,P., Piana,S. and Shaw,D.E. (2018) Developing a molecular dynamics force field for both folded and disordered protein states. *Proceedings of the National Academy of Sciences*, **115**, E4758–E4766.
21. Rauscher,S., Gapsys,V., Gajda,M.J., Zweckstetter,M., De Groot,B.L. and Grubmüller,H. (2015) Structural ensembles of intrinsically disordered proteins depend strongly on force field: A comparison to experiment. *Journal of Chemical Theory and Computation*, **11**, 5513–5524.
22. Yoo,J. and Aksimentiev,A. (2012) Improved parametrization of Li +, Na +, K +, and Mg 2+ ions for all-atom molecular dynamics simulations of nucleic acid systems. *Journal of Physical Chemistry Letters*, **3**, 45–50.
23. Barducci,A., Bonomi,M. and Parrinello,M. (2011) Metadynamics. *Wiley Interdisciplinary Reviews: Computational Molecular Science*, **1**, 826–843.

24. Barducci,A., Bussi,G. and Parrinello,M. (2008) Well-tempered metadynamics: A smoothly converging and tunable free-energy method. *Physical Review Letters*, **100**.
25. Piana,S. and Laio,A. (2007) A Bias-Exchange Approach to Protein Folding. *The Journal of Physical Chemistry B*, **111**, 4553–4559.
26. Laio,A. and Parrinello,M. (2002) Escaping free-energy minima. *Proceedings of the National Academy of Sciences of the United States of America*, **99**, 12562–12566.
27. Pietrucci,F. and Laio,A. (2009) A collective variable for the efficient exploration of protein beta-sheet structures: Application to SH3 and GB1. *Journal of Chemical Theory and Computation*, **5**, 2197–2201.
28. Case,D.A., Cheatham,T.E., Darden,T., Gohlke,H., Luo,R., Merz,K.M., Onufriev,A., Simmerling,C., Wang,B. and Woods,R.J. (2005) The Amber biomolecular simulation programs. *Journal of Computational Chemistry*, **26**, 1668–88.
29. Bonomi,M., Barducci,A. and Parrinello,M. (2009) Reconstructing the equilibrium boltzmann distribution from well-tempered metadynamics. *Journal of Computational Chemistry*, **30**, 1615–1621.
30. Kabsch,W. and Sander,C. (1983) Dictionary of protein secondary structure: pattern recognition of hydrogen-bonded and geometrical features. *Biopolymers*, **22**, 2577–2637.
31. Sridhar,A., Farr,S.E., Portella,G., Schlick,T., Orozco,M. and Collepardo-Guevara,R. (2020) Emergence of chromatin hierarchical loops from protein disorder and nucleosome asymmetry. *Proceedings of the National Academy of Sciences*, **117**, 7216–7224.
32. Schalch,T., Duda,S., Sargent,D.F. and Richmond,T.J. (2005) X-ray structure of a tetranucleosome and its implications for the chromatin fibre. *Nature*, **436**, 138–141.
33. Davey,C.A., Sargent,D.F., Luger,K., Maeder,A.W. and Richmond,T.J. (2002) Solvent mediated interactions in the structure of the nucleosome core particle at 1.9 A resolution. *Journal of Molecular Biology*, **319**, 1097–1113.
34. Apweiler,R., Bairoch,A., Wu,C.H., Barker,W.C., Boeckmann,B., Ferro,S., Gasteiger,E., Huang,H., Lopez,R., Magrane,M., *et al.* (2004) UniProt: The Universal Protein knowledge base. *Nucleic Acids Research*, **32**, 115–119.
35. Sali,A. and Blundell,T.L. (1993) Comparative protein modelling by satisfaction of spatial restraints. *Journal of Molecular Biology*, **234**, 779–815.
36. Zhou,B.R., Jiang,J., Feng,H., Ghirlando,R., Xiao,T.S. and Bai,Y. (2015) Structural Mechanisms of Nucleosome Recognition by Linker Histones. *Molecular Cell*, **59**, 628–638.
37. Humphrey,W., Dalke,A. and Schulten,K. (1996) VMD: visual molecular dynamics. *Journal of Molecular Graphics*, **14**, 33–38.
38. Schrödinger LLC (2002) The PyMOL Molecular Graphics System.
39. Onufriev,A., Bashford,D. and Case,D.A. (2000) Modification of the Generalized Born Model Suitable for Macromolecules. *Journal of Physical Chemistry B*, **104**, 3712–3720.
40. Onufriev,A., Bashford,D. and Case,D.A. (2004) Exploring Protein Native States and Large-Scale Conformational Changes with a Modified Generalized Born Model. *Proteins: Structure, Function and Genetics*, **55**, 383–394.
41. Vila,R., Ponte,I., Jiménez,M.A., Rico,M. and Suau,P. (2002) An inducible helix-Gly-Gly-helix motif in the N-terminal domain of histone H1e: a CD and NMR study. *Protein Science*, **11**, 214–220.
42. Bussi,G. (2014) Hamiltonian replica exchange in GROMACS: A flexible implementation. *Molecular Physics*, **112**, 379–384.
43. Kolinski,A. and Skolnick,J. (1997) Determinants of secondary structure of polypeptide chains: Interplay between short range and burial interactions. *Journal of Chemical Physics*, **107**, 953–964.
44. Ding,F., Jha,R.K. and Dokholyan,N. V. (2005) Scaling behavior and structure of denatured proteins. *Structure*, **13**, 1047–1054.
45. Van Zundert,G.C.P., Rodrigues,J.P.G.L.M., Trellet,M., Schmitz,C., Kastritis,P.L., Karaca,E., Melquiond,A.S.J., Van Dijk,M., De Vries,S.J. and Bonvin,A.M.J.J. (2016) The HADDOCK2.2 Web Server: User-Friendly Integrative Modeling of Biomolecular Complexes. *Journal of Molecular Biology*, **428**, 720–725.
46. McGibbon,R.T., Beauchamp,K.A., Harrigan,M.P., Klein,C., Swails,J.M., Hernández,C.X., Schwantes,C.R., Wang,L.P., Lane,T.J. and Pande,V.S. (2015) MDTraj: A Modern Open Library for the Analysis of Molecular Dynamics Trajectories. *Biophysical Journal*, **109**, 1528–1532.
